# The changing face of circulating tumor DNA (ctDNA) profiling: Factors that shape the landscape of methodologies, technologies, and commercialization

**DOI:** 10.1515/medgen-2023-2065

**Published:** 2023-12-05

**Authors:** Abel J. Bronkhorst, Stefan Holdenrieder

**Affiliations:** Technical University Munich Munich Biomarker Research Center, Institute of Laboratory Medicine, German Heart Center Lazarettstr. 36 80636 Munich Germany; Technical University Munich Munich Biomarker Research Center, Institute of Laboratory Medicine, German Heart Center Lazarettstr. 36 80636 Munich Germany

**Keywords:** Liquid biopsy, liquid profiling, cell-free DNA, circulating tumor DNA, precision medicine

## Abstract

Liquid biopsies, in particular the profiling of circulating tumor DNA (ctDNA), have long held promise as transformative tools in cancer precision medicine. Despite a prolonged incubation phase, ctDNA profiling has recently experienced a strong wave of development and innovation, indicating its imminent integration into the cancer management toolbox. Various advancements in mutation-based ctDNA analysis methodologies and technologies have greatly improved sensitivity and specificity of ctDNA assays, such as optimized preanalytics, size-based pre-enrichment strategies, targeted sequencing, enhanced library preparation methods, sequencing error suppression, integrated bioinformatics and machine learning. Moreover, research breakthroughs have expanded the scope of ctDNA analysis beyond hotspot mutational profiling of plasma-derived apoptotic, mono-nucleosomal ctDNA fragments. This broader perspective considers alternative genetic features of cancer, genome-wide characterization, classical and newly discovered epigenetic modifications, structural variations, diverse cellular and mechanistic ctDNA origins, and alternative biospecimen types. These developments have maximized the utility of ctDNA, facilitating landmark research, clinical trials, and the commercialization of ctDNA assays, technologies, and products. Consequently, ctDNA tests are increasingly recognized as an important part of patient guidance and are being implemented in clinical practice. Although reimbursement for ctDNA tests by healthcare providers still lags behind, it is gaining greater acceptance. In this work, we provide a comprehensive exploration of the extensive landscape of ctDNA profiling methodologies, considering the multitude of factors that influence its development and evolution. By illuminating the broader aspects of ctDNA profiling, the aim is to provide multiple entry points for understanding and navigating the vast and rapidly evolving landscape of ctDNA methodologies, applications, and technologies.

## 1 Introduction

The cancer genome is characterized by a heterogeneous landscape of DNA mutations, epigenetic modifications and gene expression patterns, which evolve throughout the course of the disease and in response to therapeutic pressures [1–4]. The majority of these molecular alterations are preserved in cell-free DNA (cfDNA), fragments of DNA released continuously by cancer cells, tumors and metastatic lesions into bodily fluids such as blood, urine and cerebrospinal fluid (CSF) [5–7]. Among the various sources of cfDNA, circulating tumor DNA (ctDNA) derived from tumors and present in the circulatory system is the most extensively studied and well-characterized biospecimen, and will be the main focus of this paper. In addition to conventional genomic and epigenomic features associated with cancer, e. g., DNA mutations, DNA methylation, histone modifications, and chromatin remodeling features, ctDNA molecules have been found to harbor a novel repertoire of clinically significant cancer-specific information (reviewed in [8–13]). These features include characteristics like unique end-points/motifs [14–16], fragmentation patterns [13,17–24], preferential cleavage sites [15,25,26], orientation-aware fragmentation patterns [27], and nucleosome density and spacing patterns [28,29]. Furthermore, besides mono-nucleosomal structures, ctDNA exists in many other diverse forms such as sub-nucleosomal fractions [21,30,31], high molecular weight (HMW) fragments and poly-nucleosomal fractions or “long cfDNA” [17,18,32–35], circular DNA [36–41], single-stranded DNA [30,42], protein-bound DNA [43], extracellular vesicle-associated DNA [44–46], and mitochondrial DNA [47–54], each harboring important biological and pathological information.

Given the minimally invasive nature of blood collection and the ability to sample over extended periods, ctDNA profiling has the unique potential to reconstruct real-time and temporal tumor information. Consequently, it can find utility in various aspects of cancer research and clinical oncology: (i) systematically documenting the molecular changes driving cancer; (ii) elucidating the role of these changes in tumor development, progression and therapy resistance; and (iii) serving as biomarkers for various cancer indications, either supplementing tissue biopsies or functioning as sole markers in some contexts. Numerous methods have been developed for ctDNA characterization, often tailored to specific objectives and exhibiting varying levels of analytical sensitivity and specificity. Comprehensive reviews discussing the fundamental principles, technical capabilities, clinical validity, utility of these methods, and their practical implementation, are available in refs. [5–7,9,55–60].

In this study, we pivot from intricate reviews of these topics to a lateral exploration of the vast landscape of ctDNA profiling. In its narrow definition, ctDNA profiling involves assays designed for routine clinical applications to manage cancer and its various manifestations. However, in a broader sense, the ctDNA field involves a diverse set of stakeholders (i.e., patients, students, researchers, pathologists, healthcare providers, policy makers, legal advisors, investors, drug developers, and other industry professionals), employing ctDNA profiling across various contexts (basic and translational research, clinical trials, routine tests, external quality assessments (EQAs) and ring trials), spanning many sectors (e. g., academia, diagnostic laboratories, metrology or measurement institutes, regulatory bodies, technology providers, instrument and reagent manufacturers). Collaboration among these stakeholder groups, characterized by resource sharing, technology transfer, and access to clinical samples and expertise, is crucial in enhancing and positioning ctDNA profiling as a cornerstone in personalized medicine.

This is increasingly acknowledged, as evidenced by the International Liquid Biopsy Standardization Alliance (ILSA)’s white paper. The document consolidates a plethora of programs, initiatives, and projects, presenting their developments, aims, and contributions to the ctDNA field [61]. The alliance, comprising major entities such as BloodPac, ELBS, ISLB, FNIH, BC, and NIBSC (UK), aims to cultivate a synergistic environment that encourages knowledge exchange and resource sharing, driving progress in liquid biopsy technologies. ILSA also addresses the challenges posed by the rapidly evolving field, emphasizing the need for clear communication and collaborative efforts. This important white paper, serving as both a progress update and a call for wider engagement, outlines ILSA’s collaborative initiatives aimed at harmonizing practices, accelerating advancements, and establishing standardized liquid biopsy procedures to support FDA qualification across various clinical settings.

**Figure 1:**
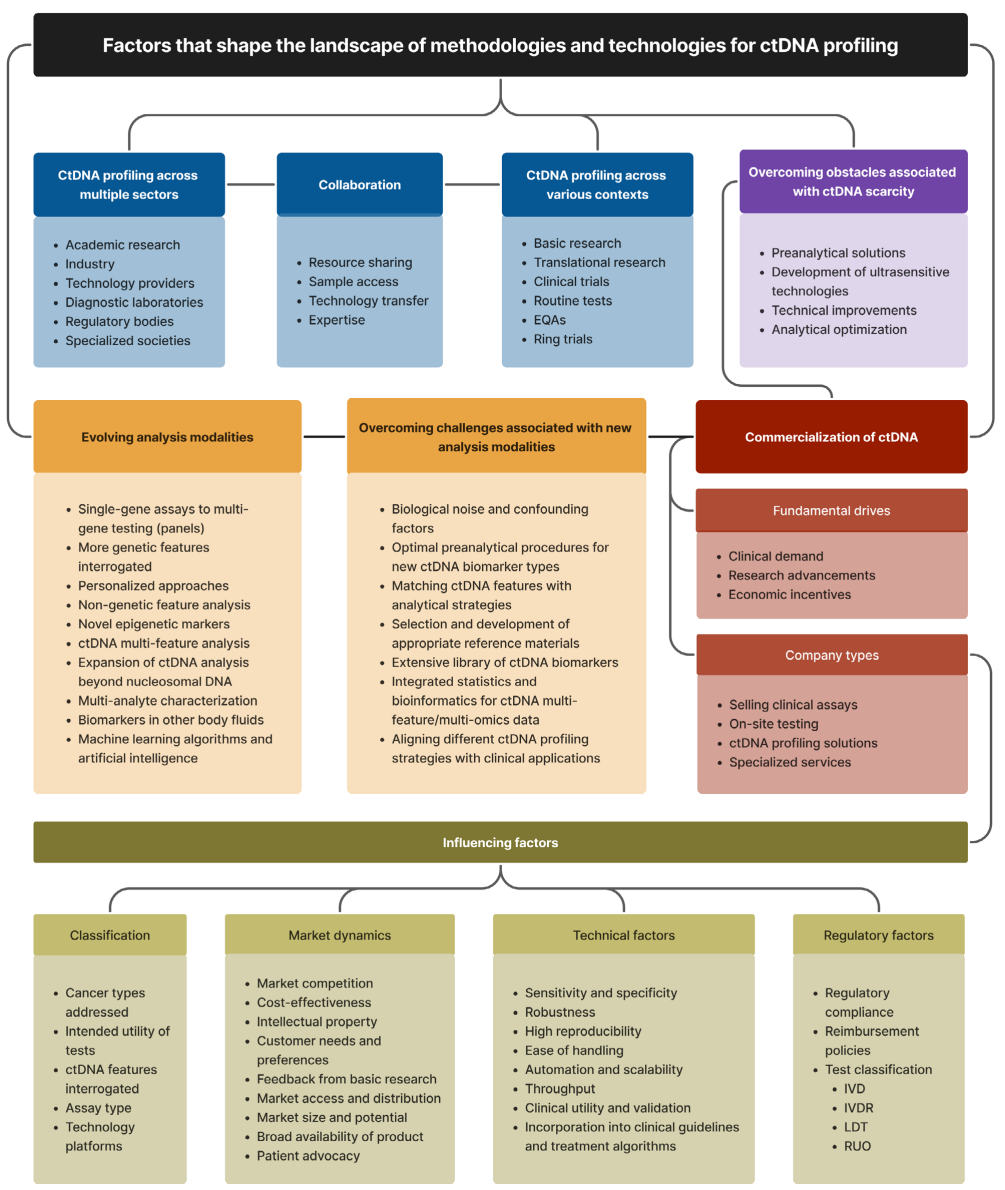
The landscape of circulating tumor DNA (ctDNA) profiling. A comprehensive overview of key factors influencing the development of ctDNA characterization methods and technologies. This categorization provides a valuable framework for assessing various aspects relevant to ctDNA research. It is important to note that certain factors may overlap between categories, as further elaborated in the text.

Despite these initiatives and the increasing interconnectivity among stakeholders, a comprehensive platform or “bridge” linking them cohesively is still absent. Moreover, current literature often does not adequately address the unique requirements and perspectives of each stakeholder group. Our paper does not attempt to bridge these substantial and complex gaps. Instead, it offers an introductory survey of ctDNA profiling, highlighting the more expansive aspects rather than dissecting the intricacies of each sector. While we delve deeper into certain key issues, our primary aim is to provide an overview or ‘roadmap’ of the primary factors influencing the ctDNA profiling and commercialization landscape, as illustrated in **Figure 1**. This roadmap is designed to serve as a starting point, offering a framework with essential reference points to assist stakeholders in navigating the field more effectively.

## 2 Factors that impact the methodological landscape of ctDNA profiling

### 2.1 Overcoming obstacles associated with ctDNA scarcity

ctDNA molecules are scarce within the prevalent cfDNA background, mainly derived from the hematopoietic system [62–64], especially mature white blood cells [65–67], notably neutrophils [68], with minor inputs from numerous other sources (reviewed in [8,69,70]). In the case of early-stage cancer, the abundance of ctDNA is often less than 1 molecule per 10 000 wild-type molecules, resulting in a variant allele frequency (VAF) or mutant allele fraction of ~0.01 % in a typical 10 mL blood specimen, with 4 mL of plasma [71]. The inherently low VAFs of ctDNA are compounded by fluctuating levels of genomic DNA in plasma, which can be influenced by diverse biological factors (reviewed in [8]). For instance, moderate exercise prior to blood withdrawal, food intake, the time of day when blood is collected, and various non-cancerous diseases and conditions can randomly or chronically increase genomic DNA levels [8,43,70,72–75]. Moreover, preanalytical errors during blood drawing and storage can inadvertently cause additional genomic DNA release [49,72,76–80].

Given these factors, achieving high sensitivity is crucial for accurate and reliable detection of ctDNA in various clinical applications, including early detection, minimal residual disease (MRD) monitoring, and assessment of treatment response. As disease progresses in many cancers, the absolute number of ctDNA molecules increases significantly, greatly enhancing the chances of their detection. The proportion of ctDNA among peripheral cfDNA varies according to tumor size [81–84], the stage of the disease [85,86] and metastatic burden [87]. However, ctDNA levels are dynamic, being influenced by a variety of other factors (reviewed in [6,8,71]). ctDNA levels fluctuate with therapy, such as chemotherapy which can induce tumor cell death, leading to increased ctDNA release. The type of tumor and its histological characteristics also influence ctDNA levels. For example, tumors with extensive vasculature can shed more ctDNA due to better access to the bloodstream. Factors within the tumor microenvironment, such as hypoxia and immune infiltration, can affect tumor cell death rates and therefore ctDNA release. Genetic diversity within tumors, rates of tumor cell death, and ctDNA clearance from the blood also contribute to ctDNA level variability. Moreover, patient-specific factors like co-morbidities, metabolism and immune function can alter ctDNA dynamics, indicating a need for nuanced approaches to ctDNA analysis. Nevertheless, in patients with advanced-stage cancer, ctDNA can exceed 10 % of peripheral cfDNA [85,88], but is much lower in patients with low tumor burden. In locally advanced disease, for example, it accounts for approximately 1 %, while in early-stage disease or after curative-intent treatment, it comprises approximately 0.1 % of total cfDNA or even less [85,89,90]. However, in addition to inherently diluted ctDNA samples, high resolution detection of ctDNA in these cases remains a challenge due to a range of issues, starting with the sequencing depth requirement. Rarely can a cfDNA sample be sequenced exhaustively, leading to a need for deeper sequencing as the VAF decreases. For instance, to recover a single mutant molecule from a sample with a VAF of 0.1 %, more than 3000x sequencing depth is necessary to mitigate stochastic issues, a standard also upheld by the 2023 guidelines of the Federal Association of Medicine in Germany (Rili-BäK). This requirement highlights the difficulties in detecting early cancer and MRD when the VAF is low. An FDA evaluation in 2021 of five commercial ctDNA assays underscored this challenge, pointing out that while these assays perform well at VAF levels above 0.5 %, their reliability falters below this threshold, resulting in inconsistent results across different vendors, labs, and assay replicates [91,92]. This inconsistency underscores the critical need for enhanced sensitivity at low VAFs. Furthermore, the inherent genetic diversity in most cancers calls for a multi-mutation analysis to ensure accurate diagnosis, necessitating a substantial amount of DNA input. In addition, critical genetic alterations, such as rare mutations and minor sub-clonal variants, may exist in very low frequencies within the ctDNA population, necessitating ultrasensitive detection methods. Comprehensive mutation analysis is also imperative, especially for applications like pan-cancer screening tests, which require analysis across a broad spectrum of mutations, further complicating ctDNA analysis. Lastly, achieving the high diagnostic sensitivity and specificity demanded by routine clinical tests often requires a considerable minimum input amount of ctDNA, despite notable advancements in analysis methods.

Various solutions have been devised to address the challenges of ctDNA analysis, encompassing preanalytical, analytical, technical, technological, bioinformatics, and biological aspects. These advancements have notably lowered the VAFs or limit of detection (LOD) for ctDNA assays and propelled the development of highly sensitive methods. Subsequent sections will delve into many of these solutions, illustrating their transformative impact on the field and highlighting areas needing further innovation.

#### 2.1.1 Preanalytical solutions

To enhance ctDNA abundance and maintain its integrity, several preanalytical strategies have been employed, with many studies documenting their efficacy in improving the precision and reliability of ctDNA assays [72,76–78,80,93,94]. These strategies encompass the use of specialized blood collection tubes like Streck™ cfDNA BCTs and PAXgene™ Blood DNA tubes, which are designed to stabilize ctDNA and avert the release of contaminating DNA from peripheral cells during sample collection and transportation [95–106]. Additionally, procedures have been developed or adopted to facilitate the collection of larger blood volumes, such as plasmapheresis, or to aggregate ctDNA from multiple blood collections (e. g., 3 × 10 mL blood collection tubes). This approach increases the probability of ctDNA detection. Enhancements in plasma storage conditions also play a crucial role, with best practices including storing plasma at –80°C and minimizing freeze-thaw cycles to prevent ctDNA degradation or loss. Moreover, processing procedures such as double centrifugation have been implemented to reduce cell lysis, limiting the release of cellular DNA and thereby improving ctDNA yield. Furthermore, the development of refined protocols for cfDNA extraction contributes to enhanced ctDNA yield and purity.

While preanalytical methods have advanced greatly, there’s room for improvement in key areas. For example, the research on optimal ctDNA storage conditions is limited, exemplified by the uncertainty surrounding the efficacy of DNA LoBind tubes, with one study noting cfDNA loss when compared to regular tubes [76]. In terms of extraction techniques, although automated ctDNA extraction offers high throughput, it often falls short in recovery efficiency compared to manual methods [107–110]. Additionally, various facets of the manual DNA extraction process, such as thawing temperature, input volume, presence of carrier RNA, use of vacuum pump-setups with spin-column kits, buffer modifications, inclusion/exclusion of denaturing agents, volume and number of elution steps, significantly impact ctDNA recovery. Despite the potential for adjustments to increase yield [76,77], these insights are frequently overlooked. It is also worth noting that current extraction processes may inadvertently introduce biases, such as favoring shorter DNA fragments or excluding longer DNA carrying crucial pathological information. Addressing these issues may require refining isolation techniques or potentially exploring extraction-free ctDNA characterization. Furthermore, capturing ctDNA based on its unique properties is a promising avenue. Identifying the physico-chemical attributes of cfDNA could lead to improved purification methods, targeting specific cfDNA subpopulations [20,111] or cancer-associated epigenetic markers [112]. However, these methods are still on the cusp of broader acceptance and commercial availability. Understanding the characteristics of cfDNA in body fluids is another complex endeavor, influenced by a myriad of biological and environmental factors. Systematically correlating these properties with various factors can unveil new biomarkers and refine cfDNA detection strategies [8,113]. For example, using a priming agent prior to blood collection has been shown to enhance cfDNA abundance by reducing cellular uptake [114]. Yet, the influence of lifestyle factors like diet or daily activity on cfDNA levels remains to be fully elucidated. Clarifying these relationships can optimize patient conditions before sample collection, thereby enhancing the detection of target molecules [72].

In a later section, we explore the practical implementation of these preanalytical solutions, shedding light on the challenges and potential pathways to optimize and standardize ctDNA preanalytics.

#### 2.1.2 Ultrasensitive methods and technologies

**Figure 2:**
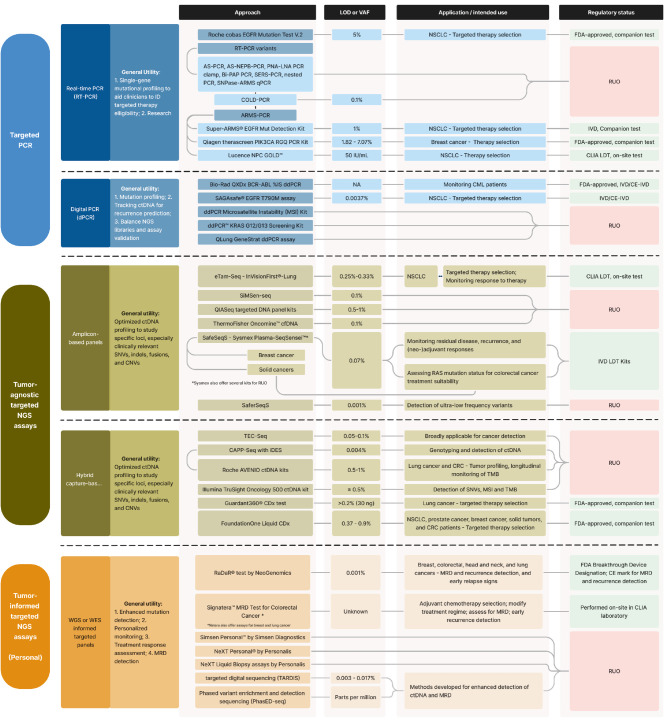
Overview of targeted assays for circulating tumor DNA (ctDNA) Analysis. This diagram presents a selection of both tumor-naïve and tumor-informed methodologies tailored for ctDNA detection, showing their respective LODs/VAFs, primary applications, and regulatory standing. A clear demarcation is made between assays designated for Research Use Only (RUO) and those that have received approval for clinical diagnostic purposes. It is important to note that this is a representative non-exhaustive catalogue, aiming to underscore the wide array of available methods and their applications. While the figure selectively features certain methods, an in-depth discussion is provided in the main text. Readers are encouraged to explore additional scientific publications and resources for a comprehensive understanding, particularly concerning the contexts in which different LODs are applicable and relevant. A similar summary for untargeted methods is provided in Figure 3.

Over time, there have been substantial advancements in methodologies and technologies for ctDNA analysis, aimed at improving sensitivity and facilitating precise reconstruction of quantitative and qualitative information from ctDNA samples (**Figure 2**). These developments have played a key role in maximizing the utility of ctDNA. The sensitivity of ctDNA assays is closely related to the VAF that they can effectively detect, which represents the proportion of mutant alleles relative to wild-type alleles in a sample. However, it is worth noting that, as the VAF depends strongly on the presence and amount of genomic DNA in plasma, some report the absolute number of mutant copies to enable better quantitative monitoring of ctDNA. Extensive reviews have been conducted to evaluate the LODs of numerous ctDNA assays across various contexts [5–7,9,55–60]. In the following sections, we provide a summary of key findings, highlighting several commercialization efforts.

##### 2.1.2.1 PCR

###### 2.1.2.1.1 Real-Time PCR (RT-PCR)

Single-gene testing, primarily through RT-PCR, has paved the way for ctDNA detection. The Roche cobas EGFR Mutation Test V.2, an RT-PCR based assay that was the first of its kind to receive FDA approval, targets 42 prevalent EGFR mutations, aiding the therapeutic selection of non-small cell lung cancer (NSCLC) patients for EGFR tyrosine kinase inhibitors (TKIs) [115]. Despite its advantages, its analytical LOD is moderate, hovering around ~5 % VAF, with significant LOD fluctuations across mutations. The inherent limitation in VAF detection of traditional RT-PCR instigated the innovation of numerous PCR-based variants (reviewed in [56,60]), such as allele-specific PCR (AS-PCR), allele-specific non-extendable primer blocker PCR (AS-NEPB-PCR), amplification refractory mutation system (ARMS) PCR, peptide nucleic acid-locked nucleic acid (PNA-LNA) PCR clamp, bidirectional-pyrophosphorolysis-activated polymerization PCR (Bi-PAP), MIDI-activated pyrophosphorolysis, SNPase-ARMS qPCR, surface-enhanced Raman spectroscopy (SERS)/PCR, multiplex PCR, nested PCR, and co-amplification at lower denaturation temperature (COLD-PCR). These variants, such as COLD-PCR that can discern VAFs as low as 0.1 %, aim to amplify sensitivity.

While most of these variants lack clinical validation, companies such as Zytomed Systems and Qiagen have developed RT-PCR assays to target specific mutations for clinical use, enhancing treatment decision-making. The Super-ARMS® EGFR Mutation Detection Kit by Zytomed Systems (through partner AmoyDx) identifies 31 somatic mutations in EGFR gene exons 18–21 from plasma samples. Designed for in vitro diagnostic (IVD) use, this kit helps determine EGFR mutation status in NSCLC patients, guiding potential EGFR-TKI treatment responses. Qiagen offers the therascreen PIK3CA RGQ PCR Kit, an IVD ARMS-PCR assay for the Rotor-Gene Q MDx instrument. It detects 11 mutations in the PIK3CA gene, aiding with identification of patients suitable for PIQRAY® (alpelisib) treatment. Those with a positive result are treatment-eligible, while further testing on FFPE tissue is recommended for negative results. The NPC GOLD™ by Lucence, an on-site RT-PCR assay, screens and monitors blood for EBV BamHI-W CpG-island DNA, enhancing early-stage Nasopharyngeal Cancer detection, thereby potentially improving survival outcomes. The test, not FDA-approved, is conducted in a CLIA-certified laboratory.

In addition to clinical applications, research-centric RT-PCR assays such as those by Biocartis, via their Idylla™ platform, have demonstrated significant alignment with next-generation sequencing (NGS) technologies: (i) the Idylla™ ctKRAS Mutation Assay, which serves as a supplement to tissue testing to identify RAS mutation status. It recognizes 21 KRAS mutations in exons 2, 3, and 4. In a study with 198 mCRC patients, it showed a 96 % match with NGS; (ii) the Idylla™ ctEGFR Mutation Assay, which identifies EGFR mutation status. It can detect 49 EGFR mutations across exons 18 to 21 and has shown a high alignment with NGS; and (iii) the Idylla™ ctNRAS-BRAF-EGFR S492R Mutation Assay, which covers 18 mutations in NRAS exons 2–4, 5 in BRAF codon 600, and 2 EGFR mutations in codon 492. In the RASANC study involving 198 mCRC patients, it displayed 100 % concordance for NRAS and 99.5 % for BRAF when compared to NGS.

###### 2.1.2.1.2 Digital PCR (dPCR)

Digital PCR (dPCR), inclusive of microfluidic-based droplet digital PCR (ddPCR) and BEAMing (beads, emulsions, amplification, and magnetics) offers a significant leap over qPCR in sensitivity for ctDNA quantification. Evaluated in a multitude of cancers, clinical indications, and bodily fluids, (reviewed in detail in [116,117]), these methods magnify traditional PCR’s sensitivity by 10–100 times, consistently detecting VAFs as low as 0.1 % to 0.01 %. Companies like Bio-Rad, SAGA diagnostics, and Biodesix have introduced assays or kits, both for research and clinical utilization, emphasizing the detection of critical mutations. The promise of dPCR in enhancing therapeutic decision-making is exemplified by FDA-approved and CE-marked kits like the Bio-Rad QXDx BCR-ABL %IS ddPCR assay, which, identifies e13a2 and e14a2 fusion transcripts, tracking p210 BCR-ABL major translocation in CML patients’ peripheral blood. Its analytical efficacy parallels the CE-IVD-marked ipsogen BCR-ABL1 Mbcr IS-MMR (Qiagen) RT-qPCR assay [118], but excludes e1a2, e19a2, and other rare transcripts. The superior sensitivity and precision of multiplexed BCR-ABL1 assays versus qPCR, combined with direct absolute quantification without standard curves, supports ddPCR’s utility in standard lab tests. SAGA Diagnostics offers a CE-IVD SAGAsafe® EGFR T790M assay utilizing their proprietary SAGAsafe® dPCR technology for detecting the EGFR p.T790M (c.2369C>T) mutation, crucial for targeted therapy in NSCLC patients with acquired EGFR TKI resistance. This assay, validated on the Bio-Rad QXDx™/QX200™ ddPCR™ system, reports a limit of blank (LOB) of 0.0010 % VAF and LOD of 0.0037 % VAF.

In terms of RUO kits, Bio-Rad offers the ddPCR Microsatellite Instability (MSI) Kit, enabling qualitative detection of deletions and insertions in microsatellite markers BAT25, BAT26, NR21, NR24, and Mono27, to be analyzed in samples from colorectal cancer (CRC) patients and other MSI-associated cancers. This method was reported to outperform a RT-PCR MSI detection test (which failed to detect any MSI) in the ctDNA of gastroesophageal adenocarcinoma patients [119]. Additionally, Bio-Rad’s ddPCR™ KRAS G12/G13 Screening Kit screens for multiple KRAS mutations, notably G12A to G12V and G13D. Biodesix presents the IQLung GeneStrat ddPCR assay, quantifying somatic genetic variants in plasma-derived circulating nucleic acids from lung cancer patients. This test detects specific somatic alterations, including insertions, deletions, point mutations, and fusions, reporting select genomic changes relevant for therapeutic decision-making. Among detected mutations in NSCLC, coverage includes EGFR (89 %), ALK (78 %), KRAS (78 %), and BRAF (54 %). It is suggested that results should be adjunctively assessed alongside patient clinical history, diagnostic tests, and clinicopathological data by a qualified healthcare professional.

###### 2.1.2.1.3 Summary

PCR technologies have been instrumental in advancing ctDNA detection. Numerous commercially available platforms and kits cater to ctDNA detection, with each varying in its input requirements and output capabilities. The diversity of commercial offerings, each with its own set of attributes, can make the selection process for a specific clinical or research endeavor quite challenging. When selecting a platform for ctDNA hotspot mutation detection, it is essential to consider criteria including test sensitivity, the scope of target coverage, peak sample throughput, and annual cost factors. Nevertheless, RT-PCR and dPCR stand out due to their proficiency in detecting ctDNA hotspot mutations. Crucially, these tools aid oncologists in treatment decisions by identifying specific mutations, facilitating the use of targeted therapies in cancers with known actionable mutations. dPCR is renowned for its heightened sensitivity in ctDNA quantification. Unlike RT-PCR, dPCR can detect a single transcript copy, providing absolute quantification without standard curves [116,118]. This superior sensitivity and accuracy have propelled dPCR to the forefront in various research and clinical applications. Despite its limited clinical integration, numerous studies indicate that dPCR effectively tracks ctDNA fluctuations, predicting recurrence even before clinical indications become apparent. The merger of dPCR with NGS offers dual benefits: understanding the tumor mutational landscape and monitoring molecular targets. Such combined insights are important for tracking treatment efficacy and predicting relapses before clinical manifestations. Beyond these uses, dPCR may be useful for optimal quantification and balancing of NGS libraries and serving as an orthogonal tool for validating NGS results. However, dPCR faces hurdles like sample size constraints, false positives arising from PCR errors, and the absence of standardized sample processing protocols. The influence of DNA purity and concentration on dPCR results warrants protocol optimization, and there are also difficulties in separating positive from negative results. However, the 2020-updated dMIQE guidelines offer a framework for streamlining dPCR experiments, accentuating key details ranging from assay creation to performance validation [116,118].

While PCR technologies are robust research tools, they face challenges. Specificity issues, such as off-target amplification and interference from non-tumor DNA, can reduce accuracy. In terms of mutation focus, PCR assays typically target known mutations, potentially missing other clinically relevant ones. Another constraint lies in the necessity for prior knowledge of mutation profiles. This limitation becomes particularly evident when dealing with tumors that have an unidentified or heterogeneous mutation landscape. Moreover, the intrinsic nature of these technologies, which relies on specific primers or probes, often curtails their multiplexing capabilities, making the simultaneous detection of multiple mutations very challenging. Lastly, PCR struggles to distinguish between mutant and wild-type alleles, affecting allele frequency evaluations and longitudinal monitoring.

##### 2.1.2.2 Next generation sequencing (NGS)

Historically, the ctDNA field faced limitations due to the singular applications of PCR-based assays. However, the emergence of NGS technologies marked a transformative moment. This shift wasn’t merely a consequence of technological capability but was instigated by a myriad of intertwined factors shaping the trajectory of ctDNA profiling. The first of these determinants lies in analytical and technical advancements surrounding NGS. Enhanced sequencing platforms now offer unparalleled throughput, improved read-lengths, and extensive sequencing depth while minimizing sequencing errors. These breakthroughs are complemented by optimized library preparation techniques and evolved sequencing chemistries. Furthermore, innovations have surged in the domain of target enrichment strategies, alongside the integration of multiplexing and barcoding approaches. These physical advancements are mirrored in the digital realm, with bioinformatics pipelines now encompassing sophisticated variant calling algorithms, stringent error correction mechanisms, rigorous quality control measures, and the holistic integration of machine learning (ML) for data interpretation. Parallel to these technical leaps is the significant influence of our expanding understanding of cancer genomics on ctDNA assay design. Assay objectives have evolved to be more diverse, spanning from targeted ones that spotlight specific genomic regions of clinical importance, to broader untargeted assays, such as whole-exome sequencing (WES) and whole-genome sequencing (WGS). Additionally, there’s an intricate layer of customization possible. While personalized assays are meticulously designed for individual patients, anchored on data from their primary tumor or baseline plasma, the field also accommodates non-personalized assays, operating without any presupposed knowledge of genetic alterations. Beyond the domains of pure science and technology, as elaborated in Section 2.5, ctDNA assay evolution is deeply intertwined with clinical and market imperatives, including regulatory landscapes, operational considerations, and economic factors, which together play key roles in integrating an assay into clinical workflows. In the subsequent sections we provide a detailed analysis of many of these major factors that have driven the development of increasingly sensitive and clinically meaningful NGS ctDNA assays.

###### 2.1.2.2.1 Tumor-agnostic targeted NGS (non-personalized assays)

Targeted sequencing is a cost-effective method to study specific loci, especially clinically relevant SNVs, indels, fusions, and copy number variations (CNVs) (reviewed in [5–7]). This approach focuses on sequencing chosen parts of the genome through target enrichment. For designing targeted assays, various resources like COSMIC, FDA-approved tests, NCCN and ASCO guidelines, OncoKB, CGI, My Cancer Genome, and Cancer Driver Log can be utilized. Assay techniques for targeted sequencing include amplicon-based or hybrid capture-based methods (reviewed in [55,57]).

**Amplicon based assays**

Amplicon-based ctDNA NGS assays use targeted amplification to detect and analyze specific regions of interest. After ctDNA extraction, a targeted amplification step employs specific primers designed to flank the regions of interest, ensuring amplification of the intended sequences. There are several amplicon-based assays available. Tam-Seq enriches genomic regions for NGS. It involves targeted preamplification, followed by precise amplification and attachment of sequencing adapters and sample-specific barcodes via optimized PCR. Tam-Seq exhibits remarkable sensitivity (>97 %) in mutation detection, even down to 2 % VAF [87]. Recent enhancements, eTam-Seq, improve its utility for highly fragmented DNA, reduce background errors, and enhance the detection of SNVs, indels, and CNVs [120]. eTam-Seq achieves exceptional performance, detecting mutations at 0.25 %–0.33 % VAF and pushing sensitivity to 0.02 %, while maintaining a high per-base specificity of 99.9997 %. Compared to dPCR, eTam-Seq demonstrates excellent concordance, underscoring its quantitative precision for low-frequency mutations [120,121]. Inivata’s InVision platform (recently acquired by NeoGenomics) utilizes eTam-Seq. Notably, InVisionFirst®-Lung is an on-site test, performed in a CLIA lab, that detects several actionable genes relevant to the treatment and management of advanced NSCLC, focusing on the identification of therapy eligibility and monitoring response to therapy.

Several companies offer amplicon-based RUO ctDNA assays. Simple, Multiplexed, PCR-based barcoding of DNA for Sensitive mutation detection using Sequencing (SiMSen-seq) [122], currently offered by Simsen Diagnostics as a service, is a method designed for creating targeted barcoded libraries using minimal DNA input and offering flexible target selection. The process is straightforward, involving a three-cycle barcoding PCR, followed by adaptor PCR for library generation, and then bead purification before sequencing. This technique permits detection of VAFs below 0.1 % and can be tailored to different library contents (from 1 to 40+ PCR amplicons). The Single Primer Extension (SPE) method, used in QIAGEN’s QIASeq targeted DNA panel kits, employs a single gene-specific primer for amplification, ensuring consistent coverage and less reliance on DNA fragment size than traditional two-primer PCR. The process begins with DNA fragmentation, which selectively avoids longer sequences. Subsequently, DNA fragments are attached to adapters containing a 12-base pair Unique Molecular Index (UMI) for amplification and minimal redundancy. SPE detects NA12878 SNPs and indels within the typical coding region with a precision range of 0.5–1 % and a 90 % accuracy rate [123]. Furthermore, the LOD for SPE extends to 1–5 % when identifying mutations across a panel of seven genes, including BRAF, EGFR, ERBB2, KRAS, NRAS, PIK3CA, and PTEN [124]. ThermoFisher Scientific offer various Oncomine™ cfDNA amplicon-based RUO assays for the detection of SNVs and hotspot indels (at an LOD of 0.1 %) in breast cancer, SNVs and short indels (0.1 %), fusions and MET exon skipping (1 %) in lung cancer, and hotspot mutations (0.1 %) in CRC. They also offer a pan-cancer 52-gene panel cfDNA assay for the detection of SNVs/short indels (0.1 %), TP53 whole-target SNVs/indels (0.5 %), and fusions and MET exon skipping (0.1 %).

A variety of NGS techniques exist for identifying rare mutations. Theoretically, NGS is capable of uncovering low-abundance mutations within a genetic sample. Yet, in practical scenarios, the intrinsic errors associated with sequencing can obscure the accurate detection of these mutations when they are present at low frequencies in the sample [125]. To address this challenge, one strategy entails employing bioinformatic analysis to calculate the probabilities that an observed mutation is more likely attributable to its presence in the original sample rather than arising as a technical artifact [126–129]. However, this approach alone often falls short of achieving the high-confidence detection required for clinical applications. As such, molecular barcoding has become crucial in enhancing the confidence of rare mutation detection in clinical applications. Safe-SeqS pioneered the use of molecular barcodes [130], assigning a unique identifier (UID) to each template molecule prior to amplification, enabling the identification of mutant PCR fragments with over 95 % identical mutations. This approach corrects amplification and sequencing errors, achieving a sensitivity of 0.05 % allele fraction. Molecular barcoding also allows for the recognition of sequencing errors by redundantly sequencing the progeny of each tagged molecule. If all progenies contain the same mutation, it is deemed genuine (a ‘supermutant’), while mutations in only a subset of progeny are considered artifacts [130]. This technology forms the basis of the Plasma-SeqSensei™ (PSS) ctDNA assays by Sysmex, which detect 0.07 % and higher VAF with 95 % accuracy amid 10 000 wild-type copies. They offer IVD kits for breast cancer to aid clinicians in monitoring residual disease, recurrence, and (neo-)adjuvant responses, and for solid cancers, including assessing RAS mutation status for CRC treatment suitability. Additionally, RUO kits are available for detecting mutations in NSCLC, melanoma and thyroid, breast, and CRCs.

Further innovations in barcoding techniques led to additional improvements in sensitivity. Molecular barcoding techniques utilize exogenous and endogenous barcodes [130]. Exogenous barcodes, consisting of specified or random nucleotides, can be added during library preparation or PCR. On the other hand, endogenous barcodes are created by the sequences at the 5′ and 3′ ends of template fragments and enable duplex sequencing [130,131]. This involves ligating a semi-degenerated double-stranded unique barcoded adapter to a target double-stranded DNA, allowing the discernment of both Watson and Crick strands based on their 5′ to 3′ directionality upon sequencing. This method significantly reduces sequencing errors, as it is unlikely for both strands to contain the same erroneous mutation generated during library preparation or sequencing [132]. The theoretical sensitivity of this approach is impressive, detecting one mutant molecule among 10^7, far surpassing conventional NGS methods [133,134]. Various molecular barcoding strategies, using endogenous or exogenous barcodes, or a combination of both, have been developed for diverse clinical applications [85,89,135–138]. A notable barcoding strategy appends identical exogenous barcodes to both Watson and Crick strands of a template molecule [133], ensuring unambiguous strand identification without relying on endogenous sequence ends and maintaining minimal error rates due to duplex sequencing.

Although this method has the lowest error rate of any sequencing technology described to date, two issues have limited its clinical applicability. First, it is challenging to convert a large fraction of the initial template molecules to adapter-ligated fragments with the same barcode on each strand [126,133,139]. This issue is particularly problematic when the amount of initial DNA is limiting, such as what is often found in liquid biopsy samples. Second, hybridization-based capture is used to enrich desired regions of the genome. While effective for enriching large regions of interest, hybridization capture does not scale well for small target regions [140] and exhibits poor duplex recovery [126,139]. Sequential rounds of capture can partially overcome these limitations, but existing hybridization capture-based methods typically recover a minority of input molecules with sequence information from both strands [139,141]. When the targeted region is very small (for example, one or a few positions in the genome of particular interest) or the amount of DNA available is limited (for example <33 ng, as is often found in plasma), capture-based approaches are suboptimal. To address these limitations, researchers introduced SaferSeqS [125], a modified version of Safe-SeqS [130]. Building on Safe-SeqS, SaferSeqS addresses library construction inefficiencies and errors. Innovations involve sequential adapter ligation to DNA fragment ends, in-situ double-stranded barcode generation (using enzymes that uniquely label DNA fragments, eliminating the need for enzymatic duplex adapter preparation that can harm DNA recovery). Limited PCR cycles create redundant copies of original DNA strands, thus a hemi-nested PCR-based approach is used for efficient enrichment, retaining strand information and recovering a high fraction of template molecules, enabling detection of low-frequency variants in limited DNA quantities. SaferSeqS demonstrates high specificity in detecting rare mutations and offers scalability, cost-effectiveness, and suitability for high-throughput automation. It reportedly outperforms existing duplex sequencing methods [126,133,134,139,141–143] with significant input recovery improvements (5- to 75-fold) and achieves over a 100-fold enhancement in error correction compared to standard PCR and ligation-based approaches using strand-agnostic molecular barcodes. SaferSeqS has two primary limitations. First, it relies on PCR-based techniques, necessitating the design of specific primers for each region of interest, which can be challenging for duplicated or hard-to-amplify regions. Second, it is most effective for analyzing relatively small genomic regions (a few to several thousand base pairs). For larger regions (exceeding 10 000 base pairs), hybrid capture methods may be more suitable. However, SaferSeqS’s duplex barcoding and library preparation components could potentially be adapted for and enhance the efficiency of hybrid capture-based duplex sequencing for larger genomic regions. As elaborated later, SaferSeqS-prepared libraries are also the foundation for a novel method known as MethylSaferSeqS. MethylSaferSeqS utilizes bisulfite-mediated deamination but retains the unique capability to detect a wide range of genetic and epigenetic alterations, all within the same DNA template [144]. This innovation holds the potential to significantly enhance the sensitivity of multi-feature ctDNA assays, particularly in situations where sample quantities are limited. These ultrasensitive methods are crucial for detecting rare mutations and MRD. Though many are not yet in clinical practice, ongoing validation studies and commercial developments are advancing these techniques.

**Hybrid capture based assays**

Hybrid capture-based ctDNA NGS assays use biotinylated oligonucleotide baits to capture specific library regions. These baits bind to target cfDNA fragments, which are then isolated using streptavidin (reviewed in [55]). Most modern hybrid capture methods use UMIs/UIDs and advanced error correction. The CAPP-Seq assay had an initial VAF detection limit of 0.02 %, identifying mutations in 100 % of stage II or higher NSCLC patients and 50 % in stage I patients [86]. By integrating barcoding with the iDES computational tool, CAPP-Seq’s sensitivity improved by 15-fold (around 0.004 % VAF) [126]. Targeted error correction sequencing (TEC-Seq), another UMI-based method, assays ctDNA for 58 genes common in several cancers and detects at least 1 mutation in over 75 % of patients at 0.01–0.1 % VAF [137]. CAPP-Seq with iDES is now available as Roche’s RUO AVENIO panels, which are pan-cancer assays but are specially optimized for lung cancer and CRC. The AVENIO ctDNA Surveillance Kit V2, is a 197 gene panel covering NCCN-guideline and emerging biomarkers, including SNVs (LOD at 0.5 %), indels (1 %), fusions (1 %) and CNVs, optimized for longitudinal monitoring of TMB. The AVENIO ctDNA Expanded Kit V2 is a 77 gene panel, and the AVENIO ctDNA Targeted Kit V2 is a smaller panel with 17 genes. The TruSight Oncology 500 ctDNA kit by Illumina is a pan-cancer panel for in-house use targeting CNVs, gene fusions, indels, SNVs, and somatic variants across 523 genes. This assay is useful for the detection of TMB and MSI. Other available RUO hybrid-capture based targeted panels include the Tempus xF Liquid Biopsy Assay (105 gene panel) and Tempus xF+ (523 gene panel) Liquid Biopsy Assay by Tempus [145], the elio™ Plasma Resolve by PGDx (now a part of LabCorp) [146], and the Guardant360 and Guardant360Response assays by Guardant Health.

In addition to numerous commercially available RUO/LDTs, the FDA recently approved two notable assays for clinical use. Guardant Health provides the FDA-sanctioned Guardant360® CDx test, which detects actionable biomarkers across 55 clinically pertinent genes, encompassing SNVs, insertions, indels, copy number alterations (CNAs) in two genes, and fusions in four genes. This test is designed as a companion diagnostic to pair lung and breast cancer patients with mutation-specific targeted treatments. Conversely, Foundation Medicine offers the expansive FDA-approved FoundationOne Liquid CDx test. It identifies substitutions and indels in 311 genes, rearrangements in four genes, and CNAs in three genes. This assay serves as a companion diagnostic to pinpoint NSCLC, prostate cancer, breast cancer, solid tumors, and CRC patients suitable for targeted therapy treatments.

In the next section we will discuss tumor-informed targeted NGS assays, which often show enhancements over tumor-naïve assays. However, a recent development worth noting here is further advancements to the CAPP-Seq assay [141]. In early-stage lung cancers, ctDNA levels are notably low but are detectable before treatment in most patients, serving as a strong prognostic indicator. Interestingly, most somatic mutations in cfDNA, both in lung cancer patients and risk-matched controls, represent clonal hematopoiesis and are not recurrent. Unlike tumor-derived mutations, those from clonal hematopoiesis appear on longer cfDNA fragments and lack the mutational signatures linked with tobacco smoking. By combining these insights with other molecular data, an ML method named ‘Lung-CLiP’ was developed and validated. This method effectively distinguishes early-stage lung cancer patients from risk-matched controls, offering performance on par with tumor-informed ctDNA detection and allowing for assay specificity adjustments for various clinical uses [141].

###### 2.1.2.2.2 Tumor-informed targeted NGS (personalized assays)

By utilizing WES or WGS on a patient’s tumor or baseline plasma, somatic variants distinct from the germline sample are identified. These detected variants enable the creation of patient-specific multiplex assays, enhancing the sensitivity of mutation detection (reviewed in [55]). These tailored panels aim to produce the maximum informative reads. In one foundational study, a plasma sample was classified as ctDNA-positive when at least two out of a median of 18 somatic variants were identified [81]. Numerous studies have presented tumor-informed sequencing panels tracking up to 20 distinct variants [86,87,126,147,148]. While these assays are resource-intensive, the increased sensitivity, along with the ability to track tumor-specific mutations facilitates personalized monitoring, treatment response assessment and MRD detection, representing a new and very promising avenue for precision medicine. There are several non-FDA-approved LDTs for the detection of MRD offered as services, some of which are currently for RUO (e. g., Simsen Personal™ by Simsen Diagnostics and NeXT Personal® and NeXT Liquid Biopsy assays by Personalis), while others are performed in CLIA labs, such as the Signatera™ MRD Test for Colorectal Cancer, Signatera™ for Breast Cancer, and Signatera™ for Lung Cancer. On the other hand, the US FDA has granted the RaDaR® test by NeoGenomics Breakthrough Device Designation for detecting MRD in early-stage cancer, and it also holds the CE mark for MRD and recurrence detection. Moreover, RaDaR is accessible to pharma, biotech firms, and commercial bodies for various cancer research stages. The RaDaR assay monitors up to 48 tumor-specific variants. Rooted in the InVision® platform, this test is designed to detect MRD, recurrence post-treatment, and early relapse signs. It is clinically validated for breast, colorectal, head and neck, and lung cancers. RaDaR employs unique algorithms to tailor panels for each patient and analyze test results, achieving an industry-leading sensitivity with a detection limit down to 0.001 % [149].

Research efforts are ongoing to improve MRD detection using tumor-informed targeted methods. As discussed earlier, patients with early-stage cancer present with low ctDNA levels compared to metastatic patients. For instance, patients with non-metastatic triple-negative breast cancer (TNBC) showed almost 20 times less ctDNA concentration before treatment compared to those with metastatic cancer [150,151]. The ctDNA signal from residual disease post-treatment is expected to be even lower, limiting the sensitivity of ctDNA tests. Challenges with sampling variation can be addressed by increasing blood volume, enhancing the DNA conversion rate for sequencing, and analyzing multiple patient-specific somatic founder mutations, which exist in all cancer cells. To enhance residual disease detection, a personalized method named targeted digital sequencing (TARDIS) was developed. TARDIS aims to boost analytical sensitivity for ctDNA analysis by deeply examining tumor-derived DNA fragments in limited plasma DNA samples. The process involves simultaneous deep sequencing of patient-specific mutations while reducing DNA loss during preparation and limiting background errors. Patient-specific somatic mutations are identified through exome sequencing of tumor biopsies. Then, numerous mutations are analyzed simultaneously in plasma DNA samples obtained during treatment. To ensure specificity and capture of input DNA, a series of procedures, including targeted linear pre-amplification, single-stranded DNA ligation with UMIs, and targeted exponential PCR, are performed. Unlike conventional PCR amplicons, this approach retains fragment size information. The use of fragment sizes and UMIs helps categorize sequencing reads and determine true mutations from potential background errors. While TARDIS showed utility for guiding treatment strategies for early-stage breast cancer, clinically relevant diagnostic threshold requires further refinement [147]. Exact Sciences recently licenced TARDIS, hoping to bring this technology to physicians and patients and deliver a better solution in MRD to improve cancer patient outcomes.

The TARDIS assay, targeting over 100 patient-specific mutations, shares a limitation common to many ctDNA assays [81,136,137]: it detects only isolated mutations, even though it analyzes data across multiple loci. This often leads to overlooking mutant signals that fail to meet the mutation calling threshold. To address this issue and improve sensitivity, the INtegration of VAriant Reads (INVAR) pipeline was developed. This pipeline consolidates sequencing reads from 10^2^ to 10^4^ mutated loci per patient and combines custom error-reduction methods with signal-enhancement techniques that leverage the biological characteristics of ctDNA. The detection limits of ctDNA are considered in terms of a two-dimensional space that stresses the importance of maximizing the number of analyzed DNA fragments. This can be achieved by either increasing the volume of plasma and number of ctDNA copies or by sampling a greater number of patient-specific variants. The count of informative reads is directly related to these two parameters. INVAR is unique in its calculation of detection limits for each sample by tallying informative reads across a range of patient-specific sites. The pipeline was utilized on 176 plasma samples from 105 patients with various cancer types and stages, demonstrating that ctDNA could be quantified to a level of 1 mutant molecule per 100 000, and in some cases, even to parts per million (ppm). The method was highly accurate, with median AUC values of 0.98 for advanced cancers and 0.80 for early-stage cancers [152] .

As discussed earlier, duplex sequencing is currently considered to be the most sensitive method for the detection of rare mutations [144]. However, unaware of the significant improvements SaferSeqS offered over other duplex sequencing approaches (likely due to overlapping publishing dates), a recent study argued against the real-world application of duplex sequencing, citing poor recovery of DNA duplexes (with only 20–25 % of all recovered molecules regaining both original strands) as a major limiting factor [126,133,153]. In response, a novel method called phased variant enrichment and detection sequencing (PhasED-seq) was developed with the aim of creating an improved MRD detection technique that simultaneously achieves low analytical detection limits and high molecular recovery for multiple mutations [153]. PhasED-seq uses multiple somatic mutations within individual DNA fragments to enhance the sensitivity of ctDNA detection. By leveraging WGS from 2,538 tumors, phased variants and their associations with mutational signatures were identified. It was demonstrated that, even without molecular barcodes, PhasED-seq outperforms prior methods, including duplex barcoding, allowing ctDNA detection in the ppm range in participant samples.

###### 2.1.2.2.3 Untargeted NGS assays

**Figure 3:**
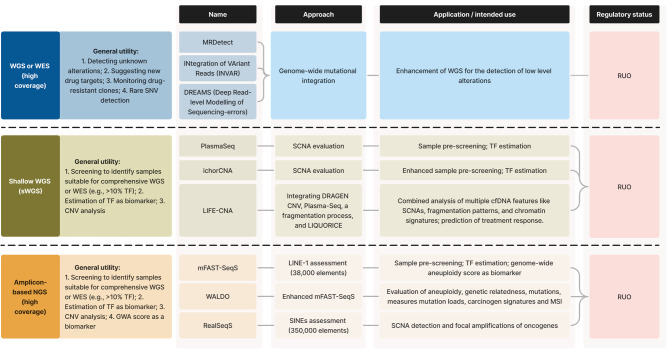
Overview of untargeted assays for circulating tumor DNA (ctDNA) Analysis. This diagram presents a selection of different untargeted approaches applicable to genome-wide analysis of ctDNA, highlighting their core principles, applications, and regulatory status. It is important to note that this is a representative, non-exhaustive list. While the figure selectively features certain methods, an in-depth discussion of various methods is provided in the main text. Readers are encouraged to explore additional scientific publications and resources for a comprehensive understanding of untargeted ctDNA assays.

Identifying single tumor DNA alterations has clinical significance, but the complexity of cancer often demands additional biological information for refining prognosis and treatment predictions. Tumor genome data, when available, can be instrumental in tracking tumor-specific changes in plasma DNA, encompassing point mutations, small Indels, structural rearrangements, and CNAs or CNVs observed in primary tumors or metastases. Targeted approaches excel at tracking these changes, making them valuable for diagnosis, monitoring residual disease, and assessing treatment efficacy. However, these methods typically rely on comprehensive characterization of the primary tumor genome, which may not always be feasible, especially with limited biopsy material, and they might not effectively monitor clonal evolution within the tumor genome. In contrast, untargeted methods do not require prior tumor genome information, making them advantageous in cases with limited or no tumor material. These methods encompass genome-wide copy number profiling and mutation spectrum evaluation, achieved through techniques like WGS, WES or extensive gene panels. While WES and WGS may be less sensitive than targeted methods [154], they afford the ability to detect previously unknown alterations crucial for initial profiling, suggesting novel drug targets, and monitoring drug-resistant clones [155]. Here we discuss prominent untargeted approaches, and how they evolved over time (**Figure 3**).

In an early WES study on ctDNA, researchers monitored six patients with advanced breast, ovarian, and lung cancers over 1–2 years. They used serial sequencing of cancer exomes in plasma samples to track genomic changes in response to therapy. The analysis of 19 plasma samples, along with synchronous biopsies, confirmed the representation of tumor genomes in plasma. Notable mutations linked to therapy resistance were identified, including mutations in PIK3CA, RB1, MED1, GAS6, and EGFR. This approach showed promise in complementing invasive biopsies for identifying drug resistance mutations in advanced cancers and offered a novel way to study clonal evolution in human cancers [156]. Several follow-up studies substantiated the potential utility of WES for detecting clinically relevant mutations in ctDNA [157–160]. However, its broader application was initially challenged by significant variations of ctDNA content, combined with a lack of clear-cut genome-wide ctDNA vs. tumor biopsy comparisons. Previous benchmarks indicated that standard-depth WES (~150× coverage) can detect somatic alterations in tumor samples with at least ~5–10 % tumor content [161], while more than 10 % is generally required for reliable determination of copy number changes [162]. However, it was recognized that, given the variability of ctDNA content across patients (0.01 % to over 90 %) [85,163–165], large-scale application of WES would require advanced screening approaches to identify samples suitable for further analysis. While previous methods focused on detecting somatic single nucleotide variants (SSNVs) in recurrently mutated cancer genes [126,166], SCNAs are more broadly applicable as most metastatic cancers exhibit arm-level SCNAs [167]. Several groups have shown that SCNAs can be detected using 0.1× WGS of cfDNA [163,164,168], also reviewed in [169]. However, methods for accurate estimation of tumor fractions (TF) required ~100-fold greater coverage [170,171]. To address this, it was hypothesized that estimating TF from 0.1× sequencing coverage could facilitate cost-effective screening for tumor-derived cfDNA in many metastatic cancer patients, thus guiding the application of WES. An analytical approach called ichorCNA was developed to quantify TF in cfDNA without prior knowledge of SSNVs or SCNAs in patients’ tumors from (ultra-low pass) ULP-WGS. ichorCNA was used to identify cfDNA samples with >10 % tumor content for WES, while subsequent analysis of WES on cfDNA and matched tumor biopsies from 41 patients demonstrated that cfDNA can effectively substitute for tumor biopsies. Further examination of 1439 blood samples from 520 patients with metastatic breast or prostate cancer using ichorCNA revealed that >30 % of blood samples and >40 % of patients had sufficient TF for standard-depth WES of cfDNA [88]. In addition to serving as a screening tool for identifying patients eligible for comprehensive cfDNA profiling, TF, estimated using the ichorCNA, has been correlated with various clinical features and responses to therapy, showing potential as a biomarker [151,172].

An alternative approach to assess the TF in cfDNA is mFAST-SeqS [162,173], which is a modification of the Fast Aneuploidy Screening Test-Sequencing System (FAST-SeqS) method [174]. In this advanced technique, a specific primer pair is used to amplify LINE1 elements scattered across the genome. This method effectively pre-screens plasma samples, providing estimates of ctDNA percentages and correlating these estimates with z-scores, which reflect the actual tumor content. Much like ichorCNA, mFAST-SeqS serves not only as a screening tool to identify suitable samples for comprehensive genomic profiling but also offers the potential of the estimated TF as a biomarker for various cancer indications. Significantly, mFAST-SeqS, with its ability to generate a genome-wide aneuploidy (GWA) score, has emerged as a promising metastatic castration-resistant prostate cancer (mCRPC) biomarker, signifying the ctDNA fraction within cfDNA. Research involving 131 mCRPC patients undergoing cabazitaxel treatment illuminated the prognostic significance of these aneuploidy scores, findings further echoed in an additional 50-patient cohort. Such revelations bolster the GWA score’s credibility as a mCRPC prognostic tool, suggesting its relevance in clinical studies centered on tumor load [175]. A similar study confirmed mFAST-SeqS’s effectiveness in assessing treatment responsiveness in mCRPC patients using GWA estimation. mCRPC patients due for treatment with androgen receptor signaling inhibitors (ARSIs) were enrolled, and blood samples were analyzed using mFAST-SeqS post-cfDNA isolation. GWA-scores, compared against healthy controls, were categorized as high or low. Results indicated shorter treatment durations in the GWA-high group versus the GWA-low among ARSI-treated patients. In essence, mFAST-SeqS-derived aneuploidy scores are key predictors of ARSI response in mCRPC patients [176].

In line with this, newly developed frameworks, such as Within-Sample AneupLoidy DetectiOn (WALDO) can improve aneuploidy detection from FAST-SeqS data. WALDO, designed for amplicon-based aneuploidy detection, excels in identifying chromosome arm changes. With ML, it streamlines GWA calls. Beyond aneuploidy detection, WALDO assesses genetic relatedness, identifies mutations, measures mutation loads, evaluates carcinogen signatures and spots microsatellite instability. Its simple procedure requires only one primer pair for PCR and amplifies 38 000 unique genomic sites, ideal for limited DNA situations. Its efficacy drops for samples with low neoplastic cell fractions, but increasing sequencing depth can improve accuracy [177]. However, due to the low genomic density of the amplicons, totaling 38 000 across the entire genome, their ability to detect focal amplifications and deletions smaller than 5 Mb was limited. Moreover, the size range of the FAST-SeqS amplicons, ranging from 120–145 bp, is suboptimal for assessing cfDNA fragments of overlapping size. Consequently, FAST-SeqS achieved aneuploidy detection in only 22 % of liquid biopsy samples containing over 1 % ctDNA. To address these limitations, researchers identified a single primer pair capable of amplifying 350 000 short interspersed nuclear elements (SINEs) with an average size of 43 bp across the genome. This development led to a significantly improved approach known as the Repetitive Element Aneuploidy Sequencing System (RealSeqS) [178]. RealSeqS was recently applied to detect cancers of the central nervous system by evaluating DNA in the CSF in 280 samples. This method assessed genome-wide copy-number alterations using WALDO and identified focal amplifications of selected oncogenes. Notably, it correctly identified 67 % of 184 cancerous and 96 % of 96 non-cancerous brain lesions. It exhibited considerably higher sensitivity compared to standard-of-care cytology and plasma cfDNA analysis in the same patient cohort. Therefore, RealSeqS has the potential to be used in conjunction with other clinical, radiologic, and laboratory-based data to facilitate the diagnosis and management of patients with suspected brain cancers [179].

Initially, sWGS was primarily used to detect CNVs, focal amplifications, chromosomal rearrangements, and aneuploidy. However, recent research suggests that sWGS can offer insights into complex tumor characteristics through multifaceted signatures. For instance, in a study involving Glioma patients, both SCNAs and DNA fragmentation patterns in CSF were analyzed. This not only identified SCNAs in 5 out of 13 patients but also revealed distinctive ctDNA fragmentation signatures in CSF. This discovery opens the door to ctDNA detection even without prior knowledge of point mutations or SCNAs within the tumor [180]. In another study, 0.5X WGS on plasma from 459 metastatic breast cancer patients, including 245 on endocrine therapy and CDK4/6 inhibitors from two cohorts, together with ML on ctDNA multi-gene signatures, detected tumor markers akin to direct tumor DNA/RNA methods. The study found four DNA subtypes and a ctDNA signature linked to retinoblastoma loss-of-heterozygosity, correlating with poor outcomes after therapy, suggesting potential broader genomic marker identification in ctDNA for various cancers [181]. In line with this, with decreasing sequencing costs, the breadth of sWGS can be increased, enabling the expansion of pipelines like Plasma-Seq, and integration of other workflows for expanded ctDNA characterization. A recent study introduced LIFE-CNA, a method to detect ctDNA in CRC patients’ plasma for diagnosis and prediction. By enhancing sWGS coverage to ~6x and integrating Illumina DRAGEN CNV, Plasma-Seq, a fragmentation process, and LIQUORICE, they combined multiple cfDNA features like SCNAs, fragmentation patterns, and chromatin signatures. Using ML classifiers, LIFE-CNA detected ctDNA with high sensitivity, predicting treatment responses 3.5 months earlier than CEA. Once validated for sensitivity and specificity, it might be adopted in clinical practice [182].

Moving beyond nuclear DNA, a recent study investigated tumor-derived cell-free mitochondrial DNA (cf-mtDNA) in liquid biopsy samples using WGS at variable coverage (0.1–30x) [183]. Using a mouse model grafted with a human CRC cell line and a study of 855 human plasma samples (655 cancer patients and 200 healthy individuals), the research highlighted the potential of tumor-derived cf-mtDNA as a biomarker for cancer. In the mouse model, increased tumor-derived mtDNA levels and distinct fragmentation patterns were observed with higher tumor load. The human study showed a median mtDNA abundance of 0.0032 %, with significant increases in certain cancer types like cholangiocarcinoma, colorectal, liver, pancreatic, and prostate cancers, but not in breast, lung, or ovarian cancers. A lack of correlation between mtDNA fraction and cancer stages was noted, and mtDNA levels were unaffected by age or gender in healthy individuals. However, variations in mtDNA fragment size distribution were observed between cancer patients and healthy individuals. Importantly, even samples with undetectable TF by ichorCNA (TF<3 %) exhibited increased mtDNA fractions compared to healthy controls, underlining the sensitivity of mtDNA as a potential early cancer detection biomarker [183].

In keeping with this, the traditional paradigm has been that WGS has utility only in the context of high-burden disease where the TF is high, and that WGS is not sufficient for the detection of low-level alterations in ctDNA and low burden disease. However, recent studies have shown that untargeted assays can also attain detection of low-level alterations. Traditional deep targeted sequencing often struggles to detect ctDNA in cases of low disease burden, primarily due to a frequently low ctDNA fraction (below 0.1 %) and a limited number of available DNA fragments in plasma samples, which can range from hundreds to thousands of genome equivalents (GEs) [184]. As a result, such sequencing can only identify ctDNA in 20–70 % of early-stage radiographically-evidenced cancer cases [81,137], and postoperative residual disease detection becomes even more challenging [148]. In response, researchers have embraced genome-wide mutational integration, which allows for the detection of ctDNA fractions as minuscule as 10^–5 at a moderate 35x sequencing depth [184]. This approach (MRDetect) benefits from WGS that can amalgamate data from different mutation types, utilize sensitivity-enhancing features like variations in ctDNA fragment lengths compared to normal cfDNA, and capitalize on nucleosome position data. All these advantages can be harvested from a singular WGS dataset, promoting both efficiency and cost-effectiveness. While MRDetect effectively detects ctDNA, it lacks robust confidence in pinpointing individual sites, such as specific driver mutations. In therapeutic scenarios demanding precise information, deeper targeted strategies would be more suitable. Nonetheless, for general cancer detection, this approach could be advantageous. Genome-wide detection usually focuses on ancestral events, those that precede cancer transformation, and is less influenced by clonal diversification. This diversification can complicate detection with custom targeted panels. Furthermore, targeted gene panels might be misled by clonal growths from hematopoietic origins, making it challenging to differentiate between age-related clonal growth and true malignancies. On the other hand, MRDetect offers high specificity, based on patient-specific mutations, maintaining accuracy even across different patient samples. Additionally, MRDetect eliminates the need to fine-tune capture panel designs and molecular noise removal, as it aligns with standard WGS. This, combined with declining sequencing costs, suggests a brighter prospect for large-scale clinical adoption. MRDetect also requires less input material (1ml of plasma) in contrast to deep sequencing strategies, which demand fully exploiting available GEs. This lower requirement is clinically vital, considering the inconsistencies in plasma and GE availability, which can affect the reliability of deep targeted methods [184]. Similar results were obtained with the INVAR pipeline. As discussed earlier, this method primarily uses previous information from tumor genotyping to guide analysis. However, with an available tumor mutation list, this method can be applied to WES and WGS, which does not require personalized sequencing panels [152].

Both above-mentioned methods involve sequencing extensive regions, making them susceptible to accumulating sequencing errors. Therefore, a specialized, custom-made solution is essential for suppressing such errors. Moreover, they prioritize combining signals from various mutations to categorize samples as either ctDNA positive or negative, rather than identifying each mutation on an individual basis. Additionally, these methods often hinge on specific curated data from known ctDNA fragments or particular lab procedures. On the other hand, an alternative strategy like DREAMS (Deep Read-level Modelling of Sequencing-errors) focuses on capturing the general patterns of read-level errors [185]. DREAMS adeptly merges features from both read levels and the local sequence context, enabling an accurate estimation of error rates, providing valuable insights for ctDNA applications. When evaluated using deep-targeted sequencing on CRC patients, DREAMS demonstrated notable potential, especially when juxtaposed with established methods such as Mutect2 and Shearwater [185].

Also addressing the limitations of targeted high coverage sequencing, a novel genome-wide method, DELFI (DNA evaluation of fragments for early interception), offers a comprehensive assessment of cfDNA “fragmentomes,” including size distribution and frequency of naturally occurring cfDNA fragments throughout the genome [16] . DELFI shows promise in detecting lung cancer with just 2x sequencing depth, making it a potential preliminary screening tool for high-risk groups. Empirical studies, like the LUCAS cohort, confirm DELFI’s ability to detect lung cancer across stages and histological variations. External validation supports its broad applicability. DELFI scores may correlate with tumor trajectory and long-term mortality, suggesting potential for uncovering hidden diseases and gauging disease severity. It can differentiate between NSCLC and SCLC, aiding non-invasive diagnosis and therapies. DELFI has identified cancer cases missed by conventional methods, emphasizing its potential in early detection, recurrence identification, and “stage shifting” in lung cancer screening. Integrating genome-wide cfDNA profiles with protein markers and clinical data could enhance sensitivity [186].

In addition to WGS and beyond the exploration of genomic alterations and DNA fragmentation, there are various untargeted approaches that bring new dimensions to ctDNA analysis. These methodologies underscore the important role of epigenetic modifications, with a special emphasis on DNA methylation, highlighting their potential as promising pathways for the management of cancer through ctDNA-based strategies (reviewed in [8]). While current techniques, such as whole genome bisulfite sequencing, may exhibit limitations [187], innovative assays like PanSeer offer hope by demonstrating precise detection of multiple cancers at early stages [188]. However, the simultaneous analysis of multiple ctDNA characteristics is challenged by the limited availability of ctDNA molecules for examination. Mitigating this challenge could potentially be achieved through the utilization of increasingly efficient commercially available library preparation protocols. Approximately 4–10 ng of cfDNA is generally applied to commonly used sWGS approaches for ctDNA analysis such as the ThruPLEX® Tag-Seq Kit (Takara) [189,190], and the TruSeq Nano DNA LT Sample preparation kit (Illumina, San Diego, CA, USA) [168]. However, newly developed library preparation kits, such as the NEBNext® Ultra™ II DNA Library Prep with Sample Purification Beads, claims good library yields with as little input as 500 picograms. Similarly, one study repurposed the Ion ReproSeq PGS Kit (Thermo Fisher Scientific), a preimplantation genetic testing kit, using only 300 pg of cfDNA for input [191]. Using this approach, the researchers were able to detect SCNAs in the cfDNA of 39 % of metastatic breast cancer patients. While promising and showing similar turn-around time to other sWGS approaches, the efficacy of this approach remains to be compared to conventional methods. In the same vein, a recent study focused on the enhanced utilization of template molecules, delving into the epigenome. This research introduced a novel method called MethylSaferSeqS, which is capable of simultaneously detecting both genetic and epigenetic alterations within the same DNA molecules. When tested on plasma samples from cancer patients, MethylSaferSeqS successfully identified expected mutations, copy number variations, and methylation patterns. Importantly, with minor adjustments, MethylSaferSeqS can be integrated with any library preparation technique that is compatible with NGS. This includes methodologies that are conducive to duplex sequencing, achieving this integration without significantly affecting the efficiency of molecule recovery [144].

Untargeted sequencing methods, despite their utility, come with several drawbacks and challenges. These methods often require a higher fraction of ctDNA, generally upwards of 5 %–10 % (and a threshold of typically ≥10 % for accurate reconstruction of tumor-specific copy number changes), posing a significant limitation. Given the high variability of ctDNA levels in blood samples, ranging from 0.01 % to over 90 %, not all samples are conducive to genome-wide analyses. Furthermore, untargeted NGS generates extensive sequencing data, requiring advanced bioinformatics tools to distinguish signals from noise, which demands computational expertise and resources. The high costs of untargeted ctDNA approaches, including sequencing, computational infrastructure, personnel, and specialized equipment, hinder universal accessibility. However, untargeted sequencing methods continue to push the boundaries of ctDNA profiling, offering insights beyond targeted counterparts. As research progresses, epigenetic analysis alongside mutational profiling may significantly enhance ctDNA profiling sensitivity and specificity, but clinical integration requires extensive validation, standardization, and evidence of clinical utility. As mentioned earlier, many commercial entities provide tumor-informed assays using WGS or WES for tumor tissue characterization. However, only a few offer comprehensive ctDNA profiling. Predicine, for instance, offers both RUO and CLIA-certified assays, such as PredicineWES+, which includes a 2500x WES and a 20 000x cfDNA panel for characterization.

### 2.2 Evolving analysis modalities

As discussed, there have been remarkable developments and changes in both core understanding and ctDNA analysis techniques over time. Detailed overviews of these advancements are available in refs. [8,113,192]. Here we provide a summary of some key transformations:

Expansion from single-gene assays to multi-gene testing (panels) – Traditionally, ctDNA analysis focused on the detection of specific genetic alterations in individual genes. However, the field has progressed towards the use of multi-gene panels, enabling the simultaneous assessment of multiple genetic targets. This approach provides a broader perspective on tumor-specific alterations and enhances the sensitivity and specificity of ctDNA-based assays (reviewed in [5–7]);
Increased scope of genetic features interrogated – The analysis of ctDNA has evolved beyond SNV analysis. Genomic approaches now encompass the investigation of various mutation classes, enabling a more comprehensive view of genomic alterations associated with cancer;
Personalized approaches – The sensitivity and specificity of variant detection, enabling a more precise and individualized assessment of the tumor characteristics and its response to treatment, has been shown to be significantly enhanced by the use of personalized assays [55];
Exploration of nongenetic features – In addition to genetic markers, ctDNA analysis now includes the evaluation of epigenetic features, such as DNA methylation patterns, histone modifications and chromatin remodeling. These features contribute to the epigenetic landscape associated with cancer development and progression. Unlike hotspot mutation profiling, many of these features occur widely across the genome, enhancing their detectability. Several of these markers are considered supplementary tests to hotspot mutational profiling, with some epigenetic assays even surpassing mutational profiling as standalone tests. Notably, the FDA-approved Epi proColon 2.0 CE test screens high-risk individuals (those over fifty years old) for CRC by detecting aberrant methylation of SEPT9 [193]. Additionally, a recent study utilizing cfMeDIP-Seq identified breast-cancer-specific methylation patterns in cfDNA from asymptomatic subjects, enabling the early detection of breast cancer years before clinical diagnosis [194]. While the diagnostic sensitivity and specificity required for clinical use have not been fully achieved, numerous studies have demonstrated strong correlations between epigenetic features of cfDNA and various indications in different cancer types [195–213]. Another advantage of epigenetic cfDNA features is the potential for simultaneous characterization of multiple cancer types. This is particularly relevant in cancer screening, where determining the tissue-of-origin is crucial. Advancements in mapping tissue-of-origin classifiers in the genome and cfDNA are making this challenging task increasingly feasible [9,214];
Identification of novel epigenetic markers -Novel epigenetic markers have emerged in ctDNA analysis. These markers include characteristics like unique end-points/motifs [14–16], fragmentation patterns [13,17–24], preferential cleavage sites [15,25,26], orientation-aware fragmentation patterns [27], and nucleosome density and spacing patterns [28,29]. These markers provide valuable insights into chromatin organization and modifications associated with tumor-specific DNA (reviewed in [8–11,113];
cfDNA multi-feature analysis – The simultaneous characterization of multiple ctDNA features has gained attention. This approach involves parallel assessment of mutations, CNVs, fragmentation patterns, and epigenetic modifications within cfDNA. Integrating multiple features enhances the accuracy and specificity of ctDNA analysis and provides a more comprehensive understanding of tumor characteristics;
Expansion of ctDNA analysis beyond mono-nucleosomal DNA – Traditional ctDNA analysis has focused on the characterization of mono-nucleosomal DNA fragments. However, the notion that mono-nucleosomal ctDNA is the sole or most relevant form of ctDNA is being challenged by evidence highlighting the importance of various other structures, including ultra-short sub-nucleosomal DNA, short poly-nucleosomal DNA (e. g., di-nucleosomes and tri-nucleosomes), HMW fragments, extrachromosomal circular DNA, neutrophil extracellular traps, DNA-protein complexes, EV-associated DNA, mitochondrial DNA, and single-stranded DNA (reviewed in [8,113]);
Integration of multi-analyte characterization – The incorporation of multiple analytes in ctDNA analysis has gained prominence [136,215–218], as they have been shown to significantly increase the sensitivity and specificity of tests. This includes the characterization of ctDNA alongside other circulating entities, such as circulating tumor cells (CTCs), EVs, proteins, miRNAs, and metabolites. The integration of diverse analytes provides a more comprehensive view of the tumor ecosystem and offers opportunities for complementary diagnostic and prognostic information;
Exploration of biomarkers in other body fluids – While ctDNA analysis has primarily focused on blood-based samples, there is increasing interest in characterizing biomarkers in other body fluids where higher target molecule concentrations are observed near the diseased tissue or organ of interest. This has shown promising results in detecting diseases such as oral, bladder, colorectal, and gynecological cancers using bodily fluids like saliva, urine, stool, CSF, cervical smears, and uterine lavage [219];
Integration of ML algorithms and AI- The incorporation of ML algorithms and AI has transformed ctDNA analysis (reviewed in [6,8,192]). These computational tools enable the identification of complex patterns, predictive modeling, and data-driven decision-making, enhancing the accuracy and efficiency of ctDNA-based diagnostics.

Collectively, these methodological advancements have revolutionized the field of ctDNA analysis, expanding our understanding of tumor biology and providing new avenues for precision oncology. Ongoing research and further innovations in these areas hold great promise for improving cancer diagnosis, monitoring treatment response, and guiding therapeutic interventions.

### 2.3 Overcoming challenges that face the new wave of ctDNA profiling

The development of ultrasensitive genetic profiling techniques for ctDNA, along with the proliferation of various ctDNA analysis methods, has established ctDNA profiling as a key component of personalized cancer care. However, this progress also brings a new spectrum of challenges and possibilities. Overcoming these challenges is essential for driving furthering advancements in the field.

#### 2.3.1 Biological noise and confounding factors

Optimal detection of ctDNA molecules is hindered by biological noise introduced by clonal hematopoiesis of indeterminate potential (CHIP), characterized by somatic mutations in blood or bone marrow cells without hematologic neoplasms, which accumulates with age and may occur in genes not typically associated with CHIP [220–223]. Detection of CHIP-associated mutations in cfDNA can lead to malignancy misdiagnosis and are observed in both cancer patients and cancer-free individuals [220–223]. Addressing noise from CHIP is increasingly crucial with higher sequencing depths and the need for detecting rare variants. The gold-standard method for CHIP filtering involves sequencing peripheral blood mononuclear cells (PBMCs) at similar or deeper depths than cfDNA [92]. However, this approach has limitations. PBMC sequencing requires a larger starting material, which poses challenges with limited samples or low blood cell counts. It yields a mixture of normal and clonal cells, potentially missing clonal mutations in cfDNA. Lack of tissue specificity in PBMC sequencing may overlook relevant clonal mutations in other cell types or tissues, leading to underestimation of the clonal burden or missing clinically significant mutations, particularly low-frequency or subclonal mutations. Moreover, the extra steps, resources, and sequencing depth requirements of PBMC sequencing limit scalability and cost-effectiveness for large-scale screening. Distinguishing between CHIP and tumor-derived variants can be aided by utilizing growing datasets, statistical analysis, and ML models. Bioinformatic approaches range from simple filtering of known CHIP genes to predictive deep learning models classifying variants as CHIP or tumor-derived [192]. Tracking tumor-informed mutations can reduce the risk of CHIP, although CHIP mutations can also be present in tumor tissue, especially in cases with low tumor purity. Another proposed approach involves differentiating CHIP-derived cfDNA from ctDNA based on fragment size differences, considering that ctDNA fragments are generally considered to be shorter [224]. However, it is important to consider the diverse cfDNA size populations in human body fluids, their overlapping origins, and increasing evidence for the presence of cancer mutations in various cfDNA size-populations.

#### 2.3.2 Preanalytical issues

As shown in Section 2.2.1, the quest for refining preanalytical conditions, methodologies, and products tailored for ctDNA analysis remains persistent. Optimizing and integrating ctDNA assays into clinical settings critically depends on wide-spread standardization and streamlining of preanalytical frameworks – keys to unlocking enhanced assay performance in terms of sensitivity, specificity, and robustness [7,49,72,76–80,94,225–227]. While commendable attempts have been made toward global alignment of standard operating procedures [7,72,93], achieving such standardization is complex, demanding careful consideration of multiple, often overlapping, challenges and variables. Here, we briefly dissect and navigate many of these complexities, outlining potential pathways towards a harmonized ctDNA preanalytical landscape.

##### 2.3.2.1 Diversity and analytical challenges of ctDNA structures

While ctDNA encompasses diverse types and structures, most current preanalytical procedures mainly target mono-nucleosomal ctDNA. However, as the importance of various ctDNA structures in signaling cancer becomes clearer, there’s a pressing need for total ctDNA standardization. The challenge lies not just in recognizing the diversity but also in developing tailored preanalytical steps that ensure integrity and optimal analysis across all ctDNA structures (reviewed in [72]). Furthermore, while preanalytical standardization for ctDNA profiling has mainly focused on blood specimens, it is essential to refine and establish specific preanalytical procedures for each bodily fluid due to the unique characteristics and composition of cfDNA molecules and inherent differences among bodily fluids in terms of stability and molecular makeup.

##### 2.3.2.2 Commercial proliferation and its consequences

The development of commercial ctDNA products is influenced by factors such as ctDNA’s genetic and epigenetic heterogeneity requiring diverse capture and analysis methods; varied laboratory methodological preferences based on considerations like expertise, ease of use and cost; advancing technology and analysis modalities pushing vendors towards innovation; demand for customization and flexibility prompting modular and tailored preanalytical systems; and the potential for unique intellectual property due to varied scientific principles. Consequently, the market witnesses an overwhelming influx of commercial ctDNA products, including multiple blood collection tubes, cfDNA extraction kits, and automated extraction platforms. While individual studies often highlight variable efficiencies among these products, a comprehensive evaluation remains elusive, complicating product selection and standardization. To navigate this terrain, a combination of independent validation efforts, centralized databases detailing product efficiencies, and a regulatory framework ensuring a baseline efficacy is vital.

##### 2.3.2.3 The complications of procedural variables

The intricacies of ctDNA preanalytics emerge further when we examine the discrepancies across studies. A meta-analysis of 20 publications on cfDNA extraction methods identified varied blood collection and processing methods, omitted procedural details, and disparate DNA quantification techniques [72]. These variances suggest that observed differences between studies might stem from overlooked procedural variables rather than the primary focus of the study. Even within the same method or product, subtle deviations as nuanced as sample-thawing temperature to buffer modifications can impact results [76]. The effect of such discrepancies become more pronounced in multi-center studies [77,79,228,229] and EQAs, as evidenced by poor concordance and high error rates [230–236].

##### 2.3.2.4 The path to standardized preanalytical protocols

Achieving preanalytical standardization is an intricate puzzle, demanding both meticulous attention to detail and collaborative efforts. Key to this is the establishment of reference metrics for each phase of the preanalytical workflow, which requires an exhaustive mapping of influencing procedural variables. Notable regulatory authorities and committees, including the German Federal Association (Rili-BäK), the European Committee for Standardization (CEN), the European Organization for Research and Treatment of Cancer (EORTC), CANCER-ID, and Bloodpac, have proposed preanalytical guidelines, but these too face challenges: varied implementation due to equipment and expertise disparities, resistance to change, the need for continuous updates owing to evolving ctDNA analysis technologies, the complexity of standardizing across diverse analysis platforms, the imperative for robust validation studies given cfDNA’s heterogeneity, and the overarching demand for global consensus to ensure harmonization. Addressing these challenges is paramount to producing effective and clear guidelines. Success in this domain promises more coherent and comparable future studies, leading to improved data quality for evidence-based guidelines. Yet, it is important to acknowledge that the pursuit of a universally applicable preanalytical workflow may be unrealistic. Instead, a nuanced approach is essential, tailoring workflows to specific contexts – be it the biospecimen type, cfDNA subtype, or the analytical modality used. Indeed, many FDA approved ctDNA assays and LDTs are routinely performed, despite the lack of global harmonization of ctDNA preanalytics. To expedite standardized preanalytics, a collaborative platform – or platforms – is crucial. This platform should: (1) promote the design of context-specific preanalytical workflows, (2) encourage detailed mapping of procedural variables and diverse protocols; (3) mandate full methodological transparency in publications, (4) facilitate sharing of methods, challenges, and solutions to align perspectives, (5) back validation of commercial product efficiency by independent labs or consortiums, (6) support a centralized database of products, highlighting their pros, cons, and applications, (7) enable the implementation of a regulatory framework for ctDNA products to ensure they surpass a set efficacy benchmark, (8) set performance metrics for each phase in a preanalytical workflow, (9) foster global collaboration among research institutes and clinical centers to share insights and to devise unified SOPs and promote their universal adoption, (10) enable the organization of workshops and training sessions to update and align researchers and clinicians on best practices; (11) consolidate all activities and insights into evidence-based guidelines with periodic updates, and (12) oversee and rectifies deviations in labs through EQAs.

The journey towards harmonized ctDNA preanalytics is paved with complexities but holds the promise of coherent, comparable studies that elevate the quality of data for evidence-based guidelines. The collaborative efforts among researchers, regulatory authorities, and independent labs will play a crucial role in shaping the future of ctDNA analysis.

#### 2.3.3 Matching ctDNA features with analytical strategies

ctDNA profiling involves numerous genetic features (e. g., SNVs, CNVs, gene fusions, indels, repetitive elements), epigenetic features (e. g., DNA methylation, histone modifications, fragment end motifs, nucleosome spacing), and structural features (size, stranded-ness, association with EVs). However, multiple methods and technical variations exist for profiling these ctDNA features, along with alternative analysis strategies (targeted vs untargeted), while different genetic and epigenetic features necessitate distinct bioinformatics procedures. Moreover, characterization of a ctDNA marker depends on the clinical context, which dictates the method sensitivity required. In addition, the field is rapidly evolving, with new methods and strategies arising continuously. This complexity makes it difficult to align a specific ctDNA feature or a set of ctDNA features with the most suitable analytical strategies. Addressing these challenges requires a combination of careful feature characterization, method selection, standardization efforts, and staying informed about the latest advancements in the field. Collaboration and sharing of knowledge among researchers, clinicians, and bioinformaticians are essential for successfully navigating these complexities.

#### 2.3.4 Selection and development of appropriate reference materials

Standard reference materials (RMs) hold a crucial role in the precision and reliability of ctDNA profiling, serving multiple critical functions [237]. First, RMs serve as benchmarks for evaluating assay performance, ensuring accuracy and dependability in results. This is achieved by comparing the outcomes from RM samples, which have established ctDNA variants and concentrations, with anticipated values. This process allows for a comprehensive assessment of the assay’s accuracy, precision, sensitivity, and specificity, thereby aiding method validation [238]. Second, the concurrent analysis of RM samples alongside patient specimens in routine clinical assessments enables continuous monitoring of key factors including assay performance, result accuracy, and the identification of any potential technical discrepancies. This practice ensures consistency and reliability in patient results, contributing to improved diagnostic confidence. Third, RMs play an indispensable role in EQAs and proficiency programs, providing a means to evaluate and ensure consistency across different laboratories [233,239]. This is crucial for determining proficiency in comparison to established benchmarks and fosters the harmonization and standardization of both preanalytical and analytical workflows across various laboratory settings.

However, despite their importance in ctDNA profiling, the integration of RMs into workflows has not been as seamless and widespread as needed. Numerous challenges associated with the use of RMs still persist and need to be systematically addressed to fully harness their potential:

Despite the availability of various types of RMs for specific ctDNA profiling applications from diverse commercial vendors (e. g., Horizon, SeraCare, anchor, SensID, Twist, ThermoFisher), comparison of RMs using different assays and platforms, followed by selection and implementation of suitable RMs are complicated by several factors, resulting in the absence of widely applicable or accepted RMs [240]. The initial major challenge is two-fold: First, while mono-nucleosomal cfDNA (~167bp) is the commonly targeted analyte in ctDNA mutational profiling assays, biospecimens are in reality genetically, epigenetically, and structurally highly diverse and poorly characterized, posing a significant challenge to creating well-matched RMs. Second, even when dealing with well-characterized cfDNA analytes (e. g., a 167 bp fragment with one SNV), many of the current production methods for cfDNA analogues are not compatible with or optimal for certain assays. For example, sonication, a popular method for simulating cfDNA fragments, generates fragments with a wide size distribution and damaged ends, resulting in reduced efficiency during sequencing library preparation. Additional challenges in developing and selecting commutable RMs arise from various factors related to the biological properties of cfDNA. These include the heterogeneity of cancer, different approaches to marker interrogation, a variety of assay methods and platforms, variability in preanalytical workflows, matrix effects, and the diversity and ctDNA concentration levels of RMs from different vendors. Each analysis necessitates careful consideration of these factors to ensure the selection of appropriate RMs. In light of these challenges, it is clear that the biological properties of cfDNA present substantial obstacles in the development and selection of suitable RMs, necessitating thorough evaluation of the specific context and requirements of each analysis.

Given these issues, it is not unexpected that various types of RMs demonstrate inconsistent performance when tested on the same platform [241]. Similarly, a singular RM can yield disparate results across different platforms [238]. This inconsistency complicates the task of discerning whether variations in detection sensitivities of methods or assays are attributable to the methodologies themselves or to the RMs in question. The importance of selecting appropriate RMs for the evaluation of liquid biopsy NGS assays is demonstrated in a recent study [241]. This study conducted a comprehensive analysis of six commercial cfDNA RMs and 42 patient samples using a duplex sequencing-based liquid biopsy NGS assay across three laboratories. To ascertain the RMs’ resemblance to native cfDNA, various metrics, including wet-lab and sequencing quality and background noise, were assessed. These factors are pivotal for both the development of liquid biopsy NGS assays and the accurate identification of true positive variants. The results showed significant variability among the RMs in terms of the evaluated metrics, with no single RM matching native cfDNA across all parameters. Based on these findings, guidelines were developed for selecting appropriate RMs at different stages of performance evaluation, highlighting the risk of reduced variant detection sensitivity when using unsuitable materials and cutoffs. The study emphasizes the need for careful RM selection in liquid biopsy NGS assay evaluation and suggests a preference for RMs with well-defined variants for assessments of sensitivity and precision, ensuring accurate clinical interpretation [241].

The solutions to the complex problems associated with RMs are not immediately clear. However, large-scale comprehensive research projects that span several sectors and expertise will be needed. One such project is the Metrology for genomic profiling to support early cancer detection and precision medicine (GenomeMET), which encompasses partners in academia, industry, metrology institutes, genomic institutes, pathology institutes, cancer institutes, and EQA providers. This project proposes a comprehensive solution aimed at enhancing genomic diagnostics across Europe. A primary objective is the development and utilization of reference measurement systems, which are specifically designed to bolster validation, quality assurance, and EQAs. In line with this, there’s a particular emphasis on reference measurement procedures (RMPs). These RMPs are tailored for cancer biomarkers and play a crucial role in determining critical Quality Control parameters within genomic workflows. Additionally, there’s a structured framework that ensures SI traceable value assignment and evaluates the commutability of RMs and EQA materials. To further enhance accuracy, there’s a robust framework established for determining Measurement Uncertainty. Lastly, GenomeMET emphasizes the importance of integrating the measurement infrastructure effectively, ensuring its wide acceptance and uptake by stakeholders in the measurement supply chain.

Moreover, as regular participation in EQA schemes is requested for laboratories conducting ctDNA diagnostics by regulatory institutions, such as the new Rili-BÄK guideline (Guideline of the Federal Medical Association), the provision of appropriate and commutable RMs with clinically relevant ctDNA concentrations will gain major interest in the future.

#### 2.3.5 Managing the extensive library of ctDNA biomarkers

The expansive range of ctDNA biomarkers available presents numerous hurdles in both the creation of ctDNA assays and the subsequent selection of suitable assays for clinical use, as outlined in **Table 1**. These challenges include increased analytical complexity, sample limitations requiring efficient utilization, potential impact on assay specificity and sensitivity, difficulties in data analysis and interpretation, varying clinical relevance of biomarkers, resource constraints, and complex validation and regulatory considerations. The selection of the right ctDNA assay for a specific clinical application necessitates careful consideration of clinical context, available resources, assay performance, and intended clinical utility. As elaborated in previous sections, collaboration among researchers, clinicians, and regulatory bodies is essential to address these challenges and optimize the development and selection of ctDNA assays that meet clinical needs effectively.

#### 2.3.6 Integrated statistics and bioinformatics for ctDNA multi-feature / multi-omics data

The integration of data from parallel characterization of multiple ctDNA features and assessment of multiple biomarkers in ctDNA profiling poses challenges in statistics, bioinformatics, computational analysis, and big data handling. Key challenges include effective data integration, dealing with complex computations and large datasets, advanced statistical analysis, application of ML and data mining, interpretation and visualization of integrated data, efficient data storage and management, addressing privacy and ethical considerations, and ensuring validation and reproducibility. These challenges remain largely unaddressed and is particularly difficult for small groups to address on their own. Collaboration among experts in various domains is essential, along with advancements in algorithms, computational resources, statistical methods, and data visualization techniques. Addressing these challenges will enhance the analysis of ctDNA profiling data and enable personalized medicine approaches with improved clinical insights.

**Table 1:** Challenges posed by an increasingly large and complex repertoire of biomarkers for ctDNA-based assays.

**Analytical challenges**	Detecting multiple biomarkers simultaneously in assays becomes technically challenging, necessitating multiplexing strategies and assay optimization.
**Sample limitations**	Limited ctDNA sample volumes necessitate efficient use and extraction methods due to the presence of numerous biomarkers.
**Specificity and sensitivity**	Increasing biomarker numbers may impact assay specificity and sensitivity due to cross-reactivity and interference, potentially resulting in false-positive or false-negative outcomes. Maintaining high specificity and sensitivity requires meticulous assay design and optimization across the biomarker repertoire.
**Data analysis and interpretation**	Extracting meaningful information from complex multi-ctDNA feature datasets becomes more challenging, requiring sophisticated bioinformatics tools and statistical methods to identify relevant patterns, correlations, and associations within the data.
**Clinical relevance**	Not all biomarkers in the repertoire may have clinical relevance for a specific application. Assessing their clinical significance requires careful consideration of biological relevance, association with disease progression or treatment response, and availability of targeted therapies or interventions.
**Cost and resource constraints**	Developing assays covering a broad ctDNA biomarker repertoire requires substantial resources. Evaluating cost-effectiveness and feasibility is crucial, considering clinical utility and resource availability in different healthcare settings.
**Validation and regulatory considerations**	Validating assays with numerous biomarkers is complex and time-consuming. Comprehensive assessment against gold standards and meeting regulatory requirements pose logistical challenges.

#### 2.3.7 Aligning different ctDNA profiling strategies with clinical applications

Profiling of ctDNA offers many potential applications throughout different stages of cancer disease management [5–7]. The two key objectives of ctDNA analyses are to either detect ctDNA with high specificity and sensitivity for cancer detection and diagnosis, or to generate a detailed tumor genome profile for various purposes such as therapy selection, risk stratification, monitoring residual disease and recurrence, assessing treatment response, and understanding treatment resistance. However, as highlighted throughout the text, selecting the most suitable ctDNA profiling strategy for a specific clinical context or question is complicated by various factors. Three major factors include (i) the required level of diagnostic sensitivity and specificity for the assay affects method and technology selection, (ii) a choice between targeted vs untargeted assays, and (iii) choice between personalized assays, tailored to an individual patient based on sequencing data obtained from the primary tumor or baseline plasma sample, or non-personalized (tumor-agnostic) assays, which are conducted without prior knowledge of alterations. Other factors include cost and turnaround time, the selection of optimal or guideline-informed biomarkers, alignment of technical requirements with available resources, various regulatory considerations and requirements (e. g., validation, compliance, ethics), and the type and quality of available samples. Finally, it is important to continuously consider emerged technologies and advancements in sequencing methodologies, molecular biology techniques, and bioinformatics tools integration.

For pragmatic reasons, it is sensible to prioritize the molecular characterization of tumor tissue obtained through biopsy or surgical resection to inform the selection of pertinent targeted or immune therapies. As advocated by the ESMO consortium [242], the profiling of ctDNA from patient plasma becomes pertinent when tumor tissue is unavailable or when an expedited therapeutic decision is imperative. This process may encompass the use of targeted panels when the tumor type and location are established and may extend to untargeted approaches when a comprehensive dataset is required. Upon the identification of hotspot mutations or other significant molecular alterations, highly sensitive targeted methodologies may be employed throughout the subsequent stages of the disease, for instance, post-surgery, during adjuvant therapy, or for MRD detection in the course of disease surveillance. The selection of these methodologies may range from ddPCR to innovative, ultra-sensitive NGS techniques, contingent upon cost considerations and the frequency of individual tests. In instances of recurrent or advanced disease, a more extensive molecular re-evaluation of the tumor is necessitated for the selection of therapy, aiming to encompass all potential druggable targets. Conversely, for ongoing monitoring of therapy response, more focused methods may suffice, capable of precisely quantifying the trajectory of mutation load in ctDNA. The emergence of novel technologies, coupled with the results from comparative analyses of various ctDNA methodologies, will increasingly clarify their integration into a comprehensive diagnostic and therapeutic framework for cancer patients. This integration aims to optimize the gleaned diagnostic information throughout the entirety of the patient’s disease journey, while maintaining cost-effectiveness.

### 2.4 CtDNA assays in the clinic: Status, development, and implementation

CfDNA sequence analysis has made significant progress in the fast-evolving field of molecular diagnostics through extensive research across various domains. The relevance and impact of cfDNA sequence analysis in clinical diagnostics is highlighted by a number of landmark developments. Firstly, the FDA has sanctioned the use of four cfDNA sequence-based tests for routine diagnostic procedures [243]. These tests facilitate the identification of druggable mutations associated with various cancers: PIK3CA mutations in breast cancer; EGFR mutations, specifically exon 19 deletions and exon 21 L858R substitution mutations, in NSCLC; KRAS G12C mutations in NSCLC; and mutations in BRCA1 and BRCA2 genes in metastatic castration-resistant prostate cancer. In addition, the FDA recently granted the RaDaR® test Breakthrough Device Designation for detecting MRD in early-stage cancer. Furthermore, an increasing number of Clinical Laboratory Improvement Amendments (CLIA) certified laboratories around the globe now provide services for profiling ctDNA mutations in cancer patients, contributing to more personalized and precise cancer care. In the realm of epigenetic analysis of cfDNA, the previously mentioned FDA-approved Epi proColon 2.0 CE test stands out as a noteworthy development [193]. The remarkable advancements and widespread research in this field underscore its potential to revolutionize diagnostics and patient care across various medical domains.

Despite its promise, however, the practical application in clinical settings is yet to reach its full potential, being applied in only a limited number of cases. As highlighted in the paper, the development of ctDNA assays that are fit for clinical roll-out is challenged by various biological, preanalytical, analytical, and technological hurdles. Another major factor is the consideration of various regulatory factors. The Evaluation of Genomic Applications in Practice and Prevention (EGAPP) set three criteria for adopting a tumor biomarker test [244]: analytical validity (test accuracy and reliability); clinical validity (the test’s ability to segregate populations based on clinical outcomes); and clinical utility (improved outcomes for tested patients) [244,245]. However, on top of these, regulation is intricate, requiring careful consideration of various factors [246]: (1) compliance with regulatory requirements, such as FDA approval or CE marking, with relevant accreditations such as CAP, CLIA, and ISO, (2) the type of tests offered, including (i) In Vitro Diagnostic (IVD) assays, which are intended for use in clinical laboratories to diagnose or guide the treatment of a specific disease. These assays have undergone rigorous regulatory approval processes and are commercially available for clinical use, (ii) In Vitro Diagnostic Regulation (IVDR) assays, which are similar to IVD assays, but they comply with the new EU regulatory framework for medical devices, (iii) laboratory developed tests (LDT), which are assays developed and performed by a single laboratory (typically CLIA-compliant), usually for research purposes or clinical testing of rare diseases. While LDTs are often reliable and need lab certification, they aren’t held to the same FDA standards, and their validity and utility might not be thoroughly vetted. However, in a significant move by the FDA, there has recently been a proposal to recalibrate the classification of IVDs, which is particularly relevant for those in the ctDNA field. This proposed amendment to the Federal Food, Drug, and Cosmetic Act (FD&C Act) underscores that IVDs, irrespective of whether they are produced by large-scale manufacturers or individual laboratories, are to be categorized as “devices”. Historically, the FDA has adopted a more lenient stance towards the enforcement of regulations on LDTs. However, the current trajectory indicates a phasing out of this enforcement discretion. Consequently, IVDs created within a laboratory environment, such as ctDNA assays, would be subjected to analogous regulatory oversight as mainstream IVDs. The rationale behind this paradigm shift is twofold: to bolster the protection of public health by ensuring the safety and efficacy of LDTs and to potentially stimulate innovation within the IVD landscape. For clarity, the term “manufacture” as employed in this context encompasses a spectrum of activities, including but not limited to design, preparation, propagation, assembly, and processing, consistent with the definitions stipulated by FDA regulations; and (iv) RUO assays, which are for research use only and are not for diagnostic or therapeutic use. It is worth noting that these categories are not mutually exclusive and that a single assay may fit into more than one category depending on its intended use, regulatory status and lab technologies applied; (3) favorable reimbursement policies, which facilitate broader adoption and utilization; and (4) incorporation into clinical guidelines and treatment algorithms, alongside integration with existing diagnostic workflows and laboratory infrastructure. Considering these factors in ctDNA methodology development and commercialization can drive successful market penetration and widespread utilization.

**Figure 4:**
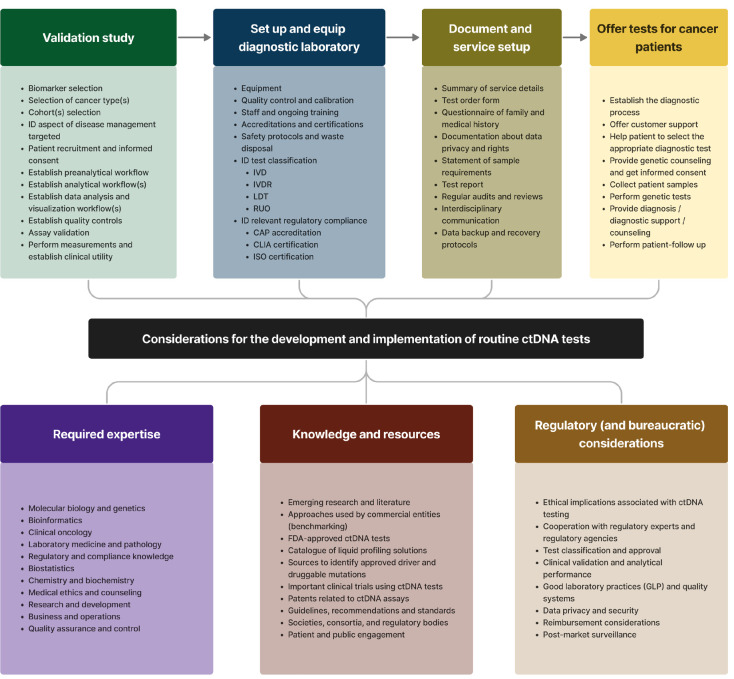
Roadmap for developing and implementing routine circulating tumor DNA (ctDNA) tests. This illustration provides a systematic approach to introducing ctDNA tests, from initial validation to patient service. Essential considerations include technical setup, documentation, service provisions, expertise requirements, pertinent knowledge resources, and regulatory factors. While this representation offers an overarching view, diving into detailed specifics is recommended for a more in-depth understanding and successful implementation.

Beyond the stipulated regulatory mandates, the formulation, validation, and deployment of either in-house (such as LDTs) or commercial ctDNA assays for accurate, dependable, and economically viable clinical routine testing necessitates a multifaceted approach. This involves rigorous planning, several procedural stages, and an array of critical considerations. While it is beyond the scope of this paper to delve into the intricacies of each factor, we provide a succinct overview of this process in **Figure 4**. It is important to note that this is a high-level summary, and more granular details should be consulted (e. g., by following the sources listed in the figure) to facilitate comprehensive understanding and implementation.

### 2.5 Commercialization of ctDNA methods, technologies, and liquid biopsy solutions

The commercialization of ctDNA-related products and services is influenced by various factors operating at multiple levels: Fundamentally, the clinical demand for improved cancer diagnosis, research advancements, and economic incentives drive the commercialization of ctDNA. As highlighted throughout the text, numerous steps in the ctDNA workflow hold significant commercialization potential. This has led to the penetration of ctDNA in diverse market domains, with the emergence of new companies and the expansion of portfolios by clinical laboratories and existing biotechnology firms, manufacturers, and distributors. These companies and institutions can be categorized broadly based on their offerings, including those selling clinical assays, offering on-site testing, providing liquid biopsy solutions (blood collection tubes, ctDNA extraction kits, etc.), or offering specialized services (e. g., data analysis, regulatory approval aid, and EQAs). Companies offering clinical tests differentiate themselves based on the addressed cancer types and intended utility of the tests (e. g., single-cancer screening, pan-cancer screening, therapy selection and guidance, risk stratification), the cfDNA features interrogated (e. g., single mutations, gene panels, DNA methylation), and the assay types and technology platforms employed.

The commercialization of ctDNA assays, propelled by competition and innovation, expedites the development and availability of vital cancer tests, enhancing patient care. However, the market’s saturation with diverse and variably efficient products complicates the selection of the optimal ctDNA profiling approach and necessitates meticulous evaluation to differentiate quality products from inferior ones. In the product and solution sector, progress is significantly driven by competition, cost-effectiveness, intellectual property, customer needs, research feedback, market access, distribution, market size, and patient advocacy. Companies innovate through competition, aiming for superior technologies and solutions. Affordability, scalability, and efficient workflows are key to cost-effectiveness, while intellectual property rights encourage research and development investments. Catering to customer needs ensures the creation of customizable and user-friendly products, with continuous feedback from research institutions aiding in product evaluation and comparison. Although not all products have been thoroughly assessed, studies have highlighted the most effective ones. Market access, distribution, and partnerships are essential for commercial success, with market size and demand for ctDNA-based diagnostics spurring investment and product innovation. Patient advocacy underscores the need for patient-focused approaches and awareness, contributing to the demand for ctDNA analysis. In clinical testing, the development and success of ctDNA products depend on market dynamics, adherence to strict criteria and regulatory compliance, favorable reimbursement, and various other factors involving technical indicators (e. g., high sensitivity, specificity, accuracy, throughput, reliable low-frequency mutation detection, robustness, reproducibility), operational aspects (user-friendliness, automation compatibility, scalability, rapid turnaround, clinical validation, quick results, and wide availability for oncological workflow integration), and economic factors (cost-effectiveness and market presence).

## 3 Concluding remarks

The characterization of ctDNA marks a transformative era in the application of molecular methods for minimally-invasive and personalized cancer patient management. However, suboptimal use, which arises from a lack of harmonization among diverse factors encompassing and influencing ctDNA profiling, is a persistent problem. In this review, we aimed to elucidate the current vast and ever-evolving methodological and technological landscape of ctDNA profiling, along with its diverse potential clinical applications. This landscape is influenced by a multitude of factors spanning multiple sectors, including academia, industry, technology providers, diagnostic laboratories, regulatory bodies, and specialized societies. Furthermore, it is shaped by various contexts, ranging from basic research and translational studies to clinical trials, routine tests, EQAs, and ring trials. Collaboration among these sectors and contexts is vital in overcoming some of the major obstacles associated with the development of clinically meaningful assays, such as ctDNA scarcity and adopting evolving analysis modalities and addressing the challenges associated with novel approaches. Moreover, the commercialization of ctDNA is influenced by various factors, emphasizing the importance of a comprehensive understanding of the dynamic interactions among these elements. Consolidating these factors and addressing inherent challenges will expedite the development of broadly accessible and impactful ctDNA tests, ultimately improving cancer diagnosis and patient care, enhancing quality of life and reducing mortality rates for cancer patients.
